# Medication Causes and Treatment of Delirium in Patients With and Without Dementia

**DOI:** 10.1002/brb3.70706

**Published:** 2025-07-21

**Authors:** Anita Elaine Weidmann, Rut Matthíasdóttir, Guðný Björk Proppé, Ivana Tadić, Pétur Sigurdur Gunnarsson, Freyja Jónsdóttir

**Affiliations:** ^1^ Department of Clinical Pharmacy Innsbruck University, Institute of Pharmacy Innsbruck Austria; ^2^ Faculty of Pharmaceutical Sciences University of Iceland Reykjavík Iceland; ^3^ Pharmacy Services Landspitali–The National University Hospital of Iceland Reykjavik Iceland

**Keywords:** delirium, dementia, medication therapy management, risk evaluation and management

## Abstract

**Introduction:**

Medication associated with delirium in adult patients is poorly understood and considered both a predisposing and precipitating factor. Prescribing advice is lacking. This systematic review aims to collate evidence on medication risks in delirium associated with causation and treatment including mechanisms, treatment, and treatment alternatives in adult patients with and without dementia in an attempt to support prescribing decision‐making and patient safety in healthcare practice.

**Methods:**

A literature review of CNIAHL, IPA, APA PsycArticles, PubMed, Cochrane Library, SAGE Education, Science Direct, SCOPUS, Web of Science Core Collection, National Grey Literature Collection, Google Scholar, Ovid, and Open Access Theses and Dissertations was performed from 2000 for articles published in English. Original, peer‐reviewed studies, meta‐analyses, systematic and narrative reviews, case reports, commentaries, editorials, and scientific communication focusing on medication‐associated delirium risk in adult patients (≥ 18 years) were included. Title, abstract, and full‐text screening were completed independently by two reviewers. CASP, MMAT, JBI, and the SANAR tool were used to assess reporting quality. The protocol was registered with the International Prospective Register of Systematic Reviews (PROSPERO) (ID: CRD42022366020).

**Results:**

Out of 3867 papers identified, 106 were included. Information about 20 different drug classes was reported. Despite a respectable quality rating, medication detail provided often lacks specificity about mechanisms of action, individual risk, dosage instructions, symptoms, and avoidable drug–drug combinations. Details on underlying mechanisms, pharmacological treatment, combinations, therapeutic alternatives, and medication associated with delirium in dementia patients were extracted.

**Conclusions:**

This summary offers the most detailed summary of medication‐related information for delirium in patients with and without dementia to support prescribing decisions. While the detailed results can be used to support a multicomponent approach to delirium care, they also support the call for categorizing delirium into distinct etiological subgroups. The effect of medication on gut microbiome diversity and composition should be considered.

## Graphical abstract

Medication associated risk related to cause and treatment of delirium

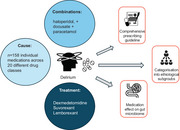

## 1 Introduction

Delirium is a complex, multidimensional neuropsychiatric illness that is common among older hospitalized patients (Wilson et al. 2020). It is characterized by an abrupt onset of disorientation and has been independently associated with a twofold increase in risk of death, a 2.4‐fold increase in risk of institutionalization, and a 12.5‐fold increase in risk of dementia (Echeverría et al. 2022; Witlox et al. 2020). Delirium can occur in patients with and without pre‐existing dementia, significantly impacting the prognosis and management of affected individuals (Inouye et al. 2014). While a plethora of patient and medical factors have been associated with delirium, medication has been considered as both a predisposing and precipitating factor (Inouye et al. 2014). The same medications that are thought to increase the risk of precipitating a delirium, such as antipsychotics and hypnotics, are used as treatment despite evidence that the use of such medications may increase the risk of mortality and prolonged hospital stays (Inouye et al. 2014). A recent meta‐analysis reported insufficient evidence for the use of antipsychotics, corticosteroids, or ketamine for the prevention or treatment of delirium. Similarly, insufficient evidence exists for anticholinergics, opioids, benzodiazepines, and H1‐antihistamines (Reisinger et al. 2023; Xu et al. 2020; Day et al. 2021; Hatta et al. 2019; Kim et al. 2020; Wang et al. 2021; The American Psychiatric Association 2024). There is a paucity of knowledge on the mechanisms of action, impact of dosage and form, risk associated with drug combinations, and suggested alternatives. There is a distinct lack of comprehensive information on medication risk in international delirium guidelines (American Psychiatric Organisation 2010). The most recently updated National Institute for Health and Care Excellence (NICE) delirium guidelines (2023) do not provide recommendations on certain medications due to a lack of high‐quality studies (National Institute for Health and Care Excellence 2023), while the American Psychiatric Association (APA) practice guidelines for the prevention and treatment of delirium (2024) suggest comprehensive medicines reconciliation and the involvement of a clinical pharmacist to manage medication risk (The American Psychiatric Association 2024).

Considering the serious negative health consequences to the patient and resultant cost to the healthcare system, comprehensive guidance is imperative to improve patient and prescribing safety. Especially, the resultant long‐term cognitive decline of patients resulting in a higher incidence of dementia is thought to cost the US healthcare system $6.9 billion (Inouye and Ferrucci 2006). Additionally, the costs incurred after hospital discharge resulting from rehospitalization, rehabilitation, home health care, or institutionalization are estimated to be over $100 billion per year (Inouye and Ferrucci 2006). Not to mention the personal cost incurred to the patient due to the functional decline and loss of quality of life (Fong et al. 2009). Especially patients with a pre‐existing diagnosis of dementia who experience delirium (delirium superimposed on dementia [DSD]) have a much higher risk of institutionalization and mortality (Han et al. 2022). The overlapping symptom profile of delirium and dementia makes DSD difficult to diagnose and is often mistaken for a sudden worsening of dementia with resultant prolonged hospital stays, increased use of hospital resources, and increased institutionalization leading to loss of independence, distress, diminished ability to engage in meaningful activities and social interactions, as well as an increased caregiver burden (Fong and Inouye 2022; Fick et al. 2005; Fong et al. 2019). A recent systematic review (SR) reports that the cost implications of delirium may be 52% higher when dementia is considered (Kinchin et al. 2021).

The aim of this extensive SR was to collate existing published evidence outside of clinical and national guidelines on medication risks in delirium, mechanisms, treatment, and treatment alternatives in adult patients with and without dementia in an attempt to support prescribing decision‐making and patient safety in healthcare practice. Clinical and national guidelines focused on psychiatry, neurology, perioperative, and pediatrics will be looked at in separate SRs (Weidmann et al. 2023, 2024, 2025; Weidmann and Tadic 2024).

## 2 Methods

**FIGURE 1:**
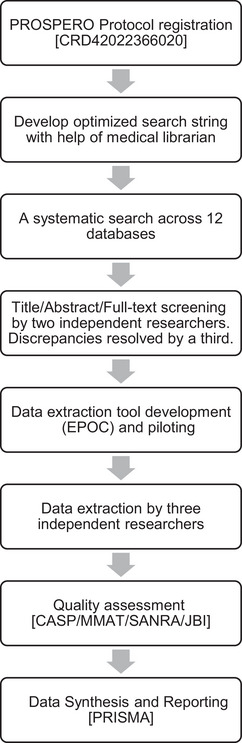
Methods flowchart summarizing the systematic stepwise approach.

An SR methodology was chosen over a scoping review as the aim required a rigorous, bias‐minimized synthesis of the existing evidence to provide definitive conclusions on medication risk in relation to the causation, prevention, and treatment of delirium. A scoping review is more suited to the identification of evidence gaps (Joanna Briggs Institute 2024). The research steps are shown in the flowchart (Figure 1).

### 2.1 Protocol and Registration

The SR protocol was registered with the International Prospective Register of Systematic Reviews (PROSPERO) (National Institute of Health Research n.d.) (CRD42022366020) and is reported following the Preferred Reporting Items for Systematic Reviews and Meta‐Analyses (PRISMA) statement (Page et al. 2021).

### 2.2 Inclusion Criteria

The Population Intervention Control Outcomes and Study Design (PICOS) criteria were used to assess study eligibility (Amir‐Behghadami and Janati 2020). Original, peer‐reviewed studies (including RCTs), narrative reviews, SRs, commentaries, editorials, and scientific communication (e.g., conference abstracts) in humans were included. All studies were focused on medication at risk of inducing or treating delirium in adult patients 18 years or older, irrespective of whether the participants had a diagnosis of dementia. The search included publications in the English language published since 2000. No geographical exclusions were applied (Table S1).

### 2.3 Searches and Study Selection

A search strategy was developed with the help of a medical librarian at Innsbruck University and verified by a medical librarian at the University of Iceland, taking various publications analyzing the language used around delirium into account (delirium [MeSH], medication therapy management [MeSH], hallucin*, altered mental status; confusion [MeSH], encephalopathy*, cognitive*; drug induced) (Puelle et al. 2015; Slooter et al. 2020). A systematic search across 12 databases was conducted (EBSCO [CNIAHL, IPA, APA PsycArticles], PubMed, Cochrane Library, SAGE Education, Science Direct, SCOPUS, Web of Science Core Collection, National Grey Literature Collection, Google Scholar, Ovid, and Open Access Theses and Dissertations). Additional manual searches of related studies listed in the references, footnotes, and citations were carried out to include any relevant additional references. All searches were re‐run prior to the final analysis. The last search for all databases was done on June 17, 2024. Only full‐text publications in the English language published since 2000 were included to capture a shift in alignment of delirium research with broader academic, clinical, and policy‐driven goals in ageing, safety, and health systems science. No unpublished studies were sought (Table S2). Database searches were managed using EndNote vs 20.6.

### 2.4 Screening

Title, abstract, and full‐text screening against the inclusion and exclusion criteria was carried out by two researchers (R.M./G.B.P.) independently, with discrepancies resolved by discussion. A third reviewer was consulted for any unresolved discrepancies (A.E.W.). During the screening process, reviewers were blinded to each other's decisions. Excel (Microsoft 365) software was used to manage the screening process.

### 2.5 Data Extraction

A data extraction tool was designed based on the Cochrane Effective Practice and Organization of Care Review Group (EPOC) checklist (Glenton et al. 2022). The tool was piloted independently by three reviewers (G.B.P./R.M./A.E.W.) on two articles. All inconsistencies were resolved by discussion, and the data extraction form was finalized. Three assessors (R.M./G.B.P./A.E.W.) independently extracted data, and any discrepancies were resolved by consultation with a fourth assessor (F.J.). The following data was extracted: title, citation, year, country of origin, study design, study aim, method, no. of patients, gender, average age of patients, medication at risk of causing delirium, mechanisms of action, contraindication, symptoms of delirium, therapeutic alternatives, information associated with medication cause of delirium in dementia patients, summary of key findings, conclusion, and limitations.

### 2.6 Outcome

The primary outcomes were mechanisms of action, dosage, form, and medication‐related information on medication in relation to the cause, treatment, or prevention of delirium. The secondary outcome was set at adult patients with a previous diagnosis of dementia.

### 2.7 Quality Assessment

The Critical Appraisal Skills Programme (CASP) Systematic Review checklist (Critical Appraisal Skills Programme 2018), the Mixed Methods Appraisal toolkit (MMAT) for quantitative non‐randomized studies (Hong et al. 2018), the Scale for Assessment of Narrative Review Articles (SANRA) for review papers (Baethge et al. 2019), and the Joanna Briggs Institute (JBI) Critical Appraisal Checklist for Case Reports (Joanna Briggs Institute (JBI) 2020) were used to conduct the quality assessment of all included papers. This was performed independently by two reviewers (R.M./G.B.P./A.E.W.) for all papers, and any discrepancies were resolved by consultation with a third (F.J.). No papers were excluded based on their quality as suggested by Dixon‐Woods et al. (2006).

### 2.8 Data Synthesis and Reporting

Medication causes of delirium along with information on mechanism of action, dose, form, and symptoms in patients with and without dementia by drug class were extracted and synthesized. Information on at‐risk combinations, suggested alternatives, and management options for antipsychotics and opioid analgesics was included. To minimize bias, extraction was undertaken independently by two researchers (R.M./I.T.), with discrepancies resolved by a third researcher (A.E.W.). Authors were not contacted for missing data. A mixed approach to synthesis was used (quantitative analysis by drug class combined with narrative synthesis for medication characteristics).

## 3 Results

The literature search resulted in 3867 publications. After removal of duplicates and independent screening, 106 publications were included in this study: *n* = 35, original research studies; *n* = 28, systematic and narrative reviews; *n* = 44, case studies; and *n* = 0, qualitative studies (Table 1). Details of the inclusion criteria (Table S1), search strategy (Table S2), PRISMA chart (Figure S1), and full reference list of publications included (Table S3) can be found in the Supporting Information.

**TABLE 1:** Summary of *n* = 106 publication characteristics and level of evidence according to Oxford Centre for Evidence‐Based Medicine (OCEBM). 1: Properly powered and conducted randomized clinical trial; systematic review with meta‐analysis – 5: Opinion of respected authorities. Case reports: (A) *n* = 35, original research studies; (B) *n* = 28, systematic and narrative reviews; (C) *n* = 44, case studies; (D) *n* = 0, qualitative studies.

(A) Original research studies				
Author (year)	Study type	*N*	Aim	OCEBM
Systematic review and meta‐analysis				
Carayannopoulos et al. (2024) [S16]	Systematic review and meta‐analysis	1750	To assess whether the use of antipsychotic medications in critically ill adult patients with delirium impacts patient‐important outcomes.	1
Wang et al. (2024) [S99]	Systematic review and meta‐analysis	5207	To comprehensively evaluate the effect of dexmedetomidine on postoperative delirium and cognitive dysfunction.	1
Han et al. (2022) [S37]	Systematic review and meta‐analysis	81,536	To elucidate the prevalence, risk factors, and impact of DSD in hospitalized older adults. Comparisons were made between older adults with DSD and persons with dementia alone (PWDs).	1
Burry et al. (2021) [S14]	A systematic review and network meta‐analysis	11,993	To compare the effects of prevention interventions on delirium occurrence in critically ill adults.	1
Ahmed et al. (2014) [S3]	Systematic review and meta‐analysis	2338	To synthesize data on risk factors for incident delirium and, where possible, conduct meta‐analysis of these.	1
Randomized controlled trials				
Bandyopadhyay et al. (2024) [S9]	Randomized controlled trial	108	To determine whether enteral melatonin decreases the incidence of delirium in critically ill adults.	1
Hatta et al. (2024) [S39]	Double‐blind, placebo‐controlled, phase 3 randomized clinical trial	203	To evaluate the orexin receptor antagonist suvorexant for reducing delirium in older adults at high risk for delirium after hospitalization.	1
Lange et al. (2024) [S59]	Randomized placebo‐controlled trial	120	To determine the effect of oral melatonin 5 mg immediate release (IR) nightly for five nights on the severity of delirium in older (≥ 65 years) medical inpatients.	1
Stollings et al. (2024) [S89]	A secondary analysis of a randomized clinical trial	566	To determine whether antipsychotics increase the QTc interval in patients with delirium in the ICU.	1
Agar et al. (2017) [S2]	Double‐blind, parallel‐arm, dose‐titrated randomized clinical trial	247	To determine the efficacy of risperidone or haloperidol relative to placebo in relieving target symptoms of delirium associated with distress among patients receiving palliative care.	1
Prospective studies				
Duprey et al. (2021) [S24]	Prospective case study	1430	To evaluate the association between ICU opioid exposure, opioid dose, and delirium occurrence.	4
Westermeyer et al. (2019) [S101]	Prospective observational study	81	To report the prevalence and recurrence rates of methadone‐related delirium, methadone doses and serum levels, causes, and treatment outcomes.	4
Tanaka et al. (2017) [S93]	Prospective study	114	To reveal the difference in the incidence of delirium between different opioids.	4
Zaal et al. (2015) [S104]	Prospective case analysis study	1112	To address these limitations and evaluate the association between benzodiazepine exposure and ICU delirium occurrence.	4
Pisani et al. (2010) [S79]	Prospective cohort study	309	To identify factors associated with persistent delirium in an older medical ICU population.	3
Sagawa et al. (2009) [S86]	Prospective case analysis study	100	To investigate the causes of delirium and their association with reversibility and motor subtypes of delirium in cancer patients.	4
Gaudreau et al. (2007) [S35]	Prospective cohort study	114	To determine whether exposure to corticosteroids, benzodiazepines, or opioids predicted delirium.	3
Gaudreau et al. (2005) [S33]	Prospective cohort study	261	To study the association between exposure to anticholinergics, benzodiazepines, corticosteroids, and opioids and the risk of delirium in cancer patients.	3
Retrospective studies				
Henmi et al. (2024) [S40]	Retrospective cohort study	710	To clarify the relationship between sleep‐inducing drugs and delirium prevention in patients hospitalized in general medical–surgical settings for nonpsychiatric conditions who underwent liaison interventions for insomnia.	3
Gale et al. (2022) [S30]	Retrospective cohort study	5131	This study has four aims: To describe the clinical characteristics associated with delirium in a population of hospital‐treated self‐poisonings; To determine which major drug classes ingested in an episode of hospital‐treated self‐poisoning are associated with delirium; To determine which drug classes, ingested in an episode of hospital‐treated self‐poisoning, are independently associated with delirium after adjustment for co‐ingestion and other confounding; To determine whether delirium in self‐poisoning patients is associated with clinically relevant outcomes: ICU admission, length of stay in the general hospital or discharge destination.	3
Herzig et al. (2022) [S41]	Retrospective cohort study	22,879	To determine the incidence and independent risk factors for potential opioid‐related ADEs within 30 days of hospital discharge.	3
Harrison et al. (2020) [S38]	Retrospective cohort study	3,311,913	To assess whether antihypertensive drug classes are associated with differential risks of delirium over a two‐year period.	3
Webber et al. (2020) [S100]	Retrospective cohort study	34,371	To describe the prevalence of delirium in patients admitted to acute care hospitals in Ontario, Canada, in their last year of life and identify factors associated with delirium.	3
Lavon et al. (2018) [S61]	Retrospective cohort study	600	To evaluate the safety of brotizolam in hospitalized patients.	3
Green et al. (2018) [S36]	Retrospective cohort study	13,627	To highlight opportunities to improve clinical decision‐making and care for patients with cognitive impairment and multiple chronic conditions.	3
Alexander Balcerac et al. (2023) [S5]	Retrospective case analysis	4559	To provide a comprehensive analysis of drug‐induced delusion, based on the World Health Organization (WHO) pharmacovigilance database.	4
Sugiyama et al. (2022) [S91]	Retrospective case analysis	259	To compare the incidence of delirium between morphine sulfate extended‐release tablets, oxycodone hydrochloride extended‐release tablets, and tapentadol hydrochloride extended‐release tablets in previously opioid‐naive patients with cancer pain.	4
Kikkawa et al. (2021) [S52]	Retrospective case analysis	2532	To investigated whether H2RAs and PPIs are risk factors for delirium, even when adjusting for other risk factors by analyzing adverse drug event reports compiled in the post‐marketing stages of drugs provided by the Japanese regulatory authorities.	4
Fick et al. (2007) [S28]	Retrospective case analysis	960	To describe the health outcomes and patterns of use of CNS‐active drugs in patients with delirium.	4
Chyou et al. (2021) [S18]	Retrospective case analysis	28,503	To identify drug combinations contributing to delirium risk in adults aged 65 and older.	4
Kassie et al. (2019) [S49]	Retrospective observational study	22,923	To assess the use of medicines associated with delirium prior to hospital admission in older Australian patients with a recorded diagnosis of delirium.	4
Hufschmidt et al. (2009) [S43]	Retrospective case analysis	346	To examine retrospectively the contribution of individual drugs to the entirety of drug‐induced acute confusional state.	4
Kolanowski et al. (2006) [S56]	Retrospective case analysis	959	To describe the pattern of antipsychotic (conventional and atypical) drug use among community‐dwelling persons with dementia.	4
Nishtala et al. (2022) [S75]	Case time control study	4818	To examine delirium risk in new users of oxybutynin and solifenacin in older adults (≥ 65 years).	4
Dyer et al. (2020) [S25]	Longitudinal relationship study	510	To assess the prevalence of sedative medication use in community‐dwelling older adults with mild–moderate AD and examine the longitudinal association between sedative medication use and adverse events (AEs).	2

**(B) Literature reviews**				
Author (year)	Study type	*N*	Aim	Quality
Maddalena et al. (2024) [S65]	Systematic review	N/A	To examine the studies on aripiprazole as a pharmacological treatment of delirium present in today's literature.	2
Tomlinson et al. (2024) [S95]	Systematic review	N/A	To describe the prevalence and patterns of antipsychotic use in delirium management with regard to best‐practice recommendations.	2
Reisinger et al. (2023) [S82]	Systematic review	N/A	To further investigate the body of evidence for drugs suspected to trigger delirium.	2
Sadlonova et al. (2022) [S85]	Systematic review	N/A	To examine the impact of diverse pharmacologic agents on delirium‐related outcomes (e.g., delirium symptom severity, duration of delirium, hospital length of stay [LOS]) in hospitalized adults.	2
Clegg et al. (2011) [S19]	Systematic Review	N/A	To summarize which medications to avoid in people at risk of delirium	2
Van Rompaey et al. (2008) [S98]	Systematic Review	N/A	To report risk factors for delirium studied in the intensive care unit.	2
Bishara et al. (2023) [S12]	Narrative review	N/A	To review the evidence for the associations of anticholinergic agents and the risk of dementia and increased mortality in dementia.	3
Korkatti‐Puoskari et al. (2023) [S57]	Narrative review	N/A	To summarize the current evidence on the risks of antipsychotic‐related falls in older adults, including aspects of pharmacokinetics and pharmacodynamics to assist clinicians in (de)prescribing antipsychotics in older people.	3
Sadlonova et al. (2023) [S84]	Narrative review	N/A	To summarize the current literature on relationships between antipsychotic medications and QTc prolongation.	3
Lauretani et al. (2020) [S60]	Narrative review	N/A	To propose a novel pharmacological flowchart of treatment in relation to the basic clusters of diseases.	3
Marvanova et al. (2016) [S67]	Narrative review	N/A	To report on cardiovascular agents that cause cognitive impairments.	3
Dharmarajan et al. (2015) [S22]	Narrative review	N/A	This review discusses antihypertensive drugs by class, including adverse effects and tolerability, with preferences in older adults and specific settings.	3
Bishara et al. (2014) [S11]	Narrative review	N/A	To identify areas of concern and which drugs to avoid in patients with dementia.	3
Anonymous (2011)	Narrative review	N/A	To summarize the potential role of pharmacotherapy in both the prevention and treatment of drug‐induced delirium.	3
Kenna et al. (2011) [S51]	Narrative review	N/A	To present the evidence for corticosteroid‐induced psychotic depression	3
Catic et al. (2011) [S17]	Narrative review	N/A	To summarize the identification and management of hospital drug‐induced delirium in older patients.	3
Kass et al. (2010) [S48]	Narrative review	N/A	To summarize the nervous system effects of antituberculosis therapy.	3
Cancelli et al. (2009) [S15]	Narrative review	N/A	To review the association between anticholinergic drug intake and the risk of developing central nervous system side effects in elderly people.	3
Jackson et al. (2008) [S46]	Narrative review	N/A	To report the adverse neuropsychiatric effects which occur with commonly used drugs in palliative care.	3
Agar et al. (2008) [S1]	Narrative review	N/A	To report on delirium in cancer patients	3
Boyle et al. (2006) [S13]	Narrative review	N/A	To provide a comprehensive review of the existing evidence‐based findings on delirium in older adults with cancer.	3
Gaudreau et al. (2005) [S33]	Narrative review	N/A	To summarize the association between psychoactive drugs and delirium.	3
Anderson et al. (2005) [S7]	Narrative review	N/A	To report on the prevention of incident delirium.	3
Zetin et al. (2004) [S105]	Narrative review	N/A	To summarize the major hazards of the new generation of psychotherapeutic drugs	3
Iqbal et al. (2004) [S44]	Narrative review	N/A	To review the therapeutic options in the management of clozapine‐induced adverse effects.	3
Alagiakrishnan et al. (2004) [S4]	Narrative review	N/A	To provide clinicians with an approach to prevent, recognize, and manage drug‐induced delirium.	3
Patten et al. (2000) [S77]	Narrative review	N/A	To report on the clinical and epidemiological evidence linking specific adverse effects to corticosteroid medications	3

A variety of research designs, were included, totaling 3,563,243 delirium patients across all 106 studies (*n* = 3,563,189, original research studies; *n* = 41, case reports; and *n* = 13, case series) of which 15,546 patients had dementia or mild cognitive impairment.

### 3.1 Mechanism of Action

The study identified several drug‐related mechanisms of action reported to increase the risk of delirium: neurotransmitter imbalances, pharmacokinetic changes, and physiological processes (Table 2).

**TABLE 2:** Summary of proposed drug‐related mechanisms of action increasing the risk of causing a delirium as stated in the literature.

Mechanism	Detail	**Supporting Information** references
Neurotransmitter imbalances	*Reduced availability*: ■ Acetylcholine ■ Melatonin	[S106, S60, S30, S12, S5, S4, S12, S85, S26, S71, S76, S4, S34, S13, S29, S98, S15, S67]
	*Increased availability*: ■ Dopamine (agonists and dopamine precursors) ■ Norepinephrine (agonists and precursors) ■ Glutamate (agonist) ■ NMDA receptor (agonist) ■ Increased blood–brain barrier permeability (permeability‐glycoprotein P‐gp)	
	*Variable alterations*: Serotonin (agonists or antagonists) Histamine (agonists or antagonists) Gamma‐aminobutyric acid (GABA)‐A (agonists or antagonists) Somatostatin (agonists or antagonists) Norepinephrine (agonists or antagonists) Endorphins (agonists or antagonists)	
	*Inhibition* Neuronal NA^+^/K^+^ ATPase Muscarininc receptors (antagonists/blockers)	
Pharmacokinetic changes	Reduction in *gastric acid secretion*, gastric emptying time, gastrointestinal motility, blood flow to internal organs, and absorptive surface area *Doubling of fat* content and decreasing of intracellular water, increasing the volume of lipid‐soluble drugs *Reduced albumin* concentrations, increasing risk of drug toxicity due to higher amounts of select unbound drug plasma concentration *Diminished hepatic metabolism* with a subsequent decline in the cytochrome P450 function *Reduced renal function* resulting in increasing risk of drug toxicity due to delayed drug clearance	[S12, S5, S4, S12, S77, S13, S98, S67]
Physiological factors	Neuro‐inflammation Oxidative stress Neurodegeneration Sleep dysfunction Neurotransmitter diurnal dysregulation Hyponatremia Acid/base imbalance Fluid/electrolyte imbalance	[S60, S12, S4, S60, S13, S98, S86]

### 3.2 Medications at Risk of Causing a Delirium

**FIGURE 2:**
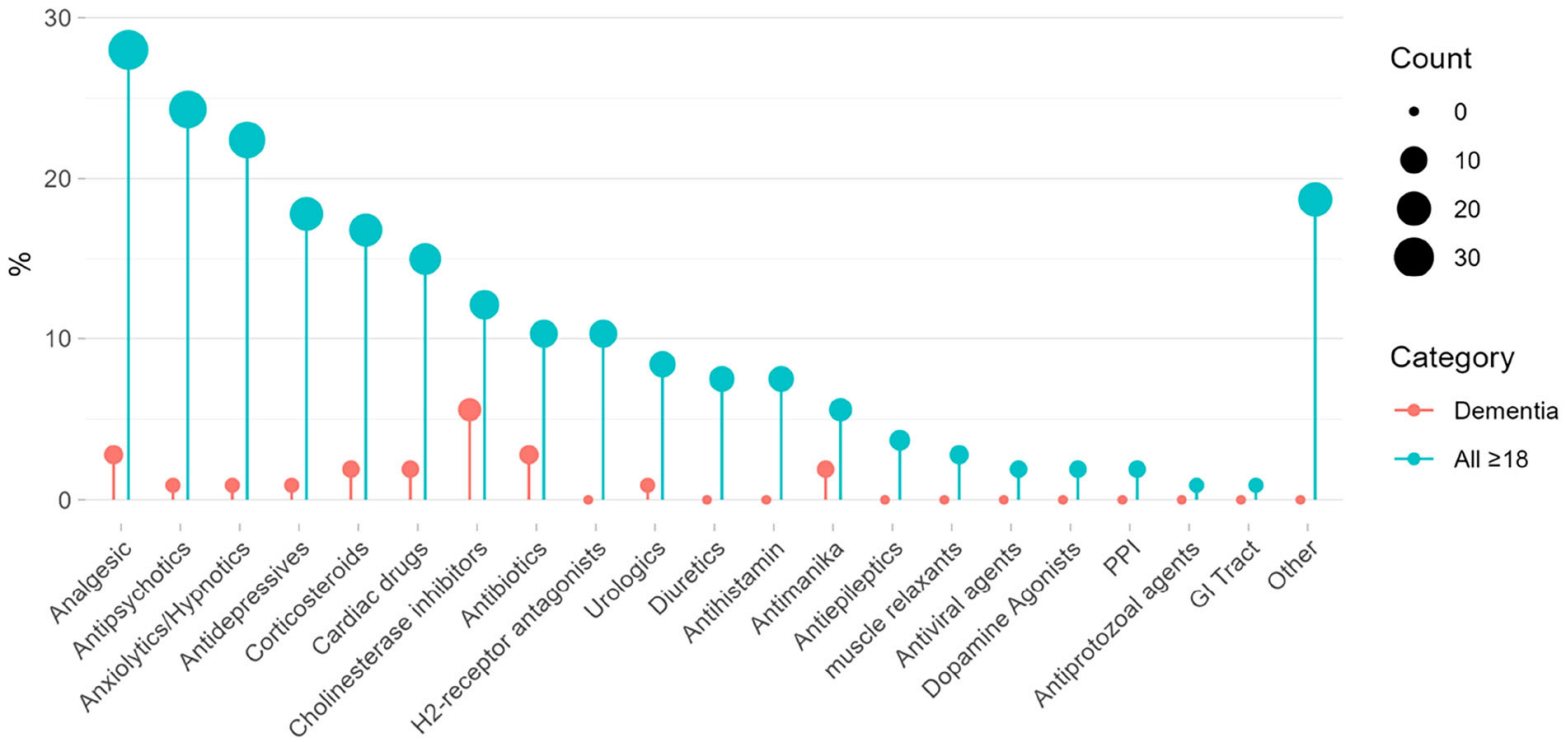
Reporting frequency of 20 different drug classes associated with an increased risk of delirium with and without dementia across all included publications (*n* = 106). Please note that this table is at risk of publication bias.

A total of *n* = 158 individual medications were identified across 20 different drug classes (Figure 2 and Figure S2). The most frequently mentioned medication classes opioid analgesics (*n* = 24), antipsychotics (*n* = 23), anxiolytics/hypnotics (*n* = 19), antidepressants (*n* = 18), corticosteroids (*n* = 16), cardiovascular (*n* = 16), cholinesterase inhibitors (*n* = 13), and antibiotics (*n* = 11) are described in this Section 3. Less frequently mentioned medication detail are shown in Table S4.

#### 3.2.1 Opioid Analgesic

A total of *n* = 22 papers (20.7%) suggest that opioid analgesics are a substantive risk factor for delirium (Tables 3 and 4) (see references [S4, S5, S8, S10, S11, S13, S17–S19, S24, S35, S41, S45, S46, S53, S64, S78, S79, S91, S93, S98, S101] in the Supporting Information). Mechanisms such as an altered blood–brain barrier integrity, facilitating the entry of pro‐inflammatory cytokines and toxins, as well as the apoptosis of human neurons and microglial cells, through the upregulation of Bax and downregulation of the anti‐apoptotic protein Bcl‐2 are considered to be responsible for the opioid‐associated cognitive impairment (Cunha‐Oliveira et al. 2008). An equivalent dose of > 54 mg/day was reported to be a factor significantly associated with persistent delirium [S79], while the odds ratio for morphine daily doses of 7.2–18.6 mg (OR = 9.20) was associated with a significant increase in the risk of developing delirium among intensive care (ICU) patients [S98]. The incidence of delirium was 4.8% (*n* = 83) for morphine in an observational study of 259 opioid‐naive oncology inpatients [S91]. However, there was no detectable significant difference in the incidence of delirium between morphine, oxycodone, or tapentadol hydrochloride [S91]. The administration of intravenous morphine equivalent dose was associated with a 2.4% increased risk of delirium, as was epidural ropivacaine hydrochloride use during anesthesia [S91]. While it is most difficult to manage delirium/hallucinations or somnolence induced by transdermal fentanyl, characterized by its long half‐life of 6–7 h, oxycodone has the shortest half‐life (*t*_1/2_ = 3 h), resulting in a more predictable dose–response relationship [S10, S11]. It is noteworthy that dose levels of > 80 mg methadone were associated with the precipitation of a delirium. This risk doubled in doses above 150 mg and doubled again in doses above 200 mg/day [S101].

**TABLE 3:** Summary of reported drug‐specific mechanism, dosing, and symptom information for the most frequently mentioned drugs and drug classes. Only drugs for which enough information was available were include.

Drug class	Drug [ATC code]	Mechanism of action	CYP p450	Dose	Symptom	Comments	**Supporting Information** references
Opioid analgesic	General information	Mitochondrial dysfunction, oxidative stress, and upregulation of apoptogenic proteins, which can produce permanent white matter lesions	—	Higher opioid doses increase the risk Cumulative daily doses of opioids above 90 mg	Hallucination	Similar risk of delirium in opioid‐naive patients among morphine, oxycodone, and tapentadol oral opioids Delirium is the most common potential opioid‐related ADE	[S11, S18, S35, S41, S79, S91, S93, S101]
	Buprenorphine [N02AE01; N07BC01]	Dopamine‐releasing effect	CYP3A4 and CYP2D6 inhibitor	—	Inactivity and lethargy	■ Transdermal application increases risk ■ IV opioids carry the highest risk of mortality	[S45]
	Fentanyl [N01AH01; N02AB03]	Possible muscarinic receptor binding	CYP3A4 inhibitor	Dose‐dependent toxicity	Hallucination somnolence	■ Transdermal application increases risk ■ IV opioids carry the highest risk of mortality	[S10, S18, S19, S46]
	Morphine [N02AA01]	Inhibition of GABA receptors allows dopaminergic neurons to fire more vigorously	CYP2D6 inducer	> 54 mg/day PO associated with persistent delirium Each daily 10‐mg IV morphine‐equivalent dose was associated with a 2.4% increased risk for delirium the next day	—	■ Extended release tablets increases risk ■ IV opioids carry the highest risk of mortality	[S98, S46, S91, S24]
	Oxycodone [N02AA05]	Reduction of GABAergic inhibition of dopamine neurons, leading to an increase in dopamine production and release	CYP2D6	—	Delusion	■ Oxycodone is associated with a lower risk for delirium compared to other opioids	[S5, S53, S64, S91]
	Pethidine [N02AB02]	Inhibition of the dopamine and norepinephrine transporter	CYP2B6 substrate	—	—	■ Pethidine was associated with a higher risk of causing delirium than oxycodone	[S4, S11, S13, S17, S19, S78]
	Methadone [N07BC02]	Affects the release of acetylcholine, norepinephrine, substance P, and dopamine	CYP3A4 substrate CYP2B6 substrate CYP2D6 inhibitor	> 80 mg; risk doubled above 150 mg and doubled again above 200 mg/day	—	■ Previous methadone‐related delirium predicted recurrence	[S101]
	Tramadol [N02AZ03]	Inhibits norepinephrine, serotonin, and dopamine	CYP2D6 substrate	—	Confusion psychotic symptoms, violent behavior delusion	■ Tramadol IM induces delirium ■ Only use in older adults after careful consideration	[S8, S17, S78]
Antipsychotics	General information	High anticholinergic burden	—	—	—	■ Antipsychotics are a highly possible risk factor for delirium. ■ Moderate degree of QTc prolongation ■ Meta‐analysis found that antipsychotic use was not associated with changes in the duration or severity of delirium ■ Only use unless patient is at risk of harm to self and others [Beers/SToppFall] ■ All antipsychotic use for delirium and insomnia is therefore off‐label ■ Dementia may predispose patients to ACH drug‐induced delirium	[S12, S46, S57, S72, S84]
	Haloperidol [N05AD01]	D2 receptor antagonist	CYP3A4 substrate CYP2D6 inhibitor	IV haloperidol Haloperidol (treatment)—use 1 week or less	Delirium, neuroleptic malignant syndrome	■ Conflicting evidence of causal risk for delirium ■ IV haloperidol increased risk of QTc prolongation (medium–high risk) ■ Haloperidol + omeprazole—avoid ■ Co‐administration with carbamazepine leads to a significant change in the pharmacokinetics of haloperidol ■ Haloperidol may negatively affect survival compared to placebo	[S2, S4, S13, S17, S18, S31, S60, S65, S79, S82, S84, S89, S95]
	Olanzapine [N05AH03]	D2 receptor antagonist	CYP1A2 substrate	PO, IM, and IV olanzapine as safe and effective treatments for critically ill patients with delirium	Impaired consciousness and attention, agitation visual hallucination	■ Reports are rare ■ Fluvoxamine increases the plasma concentrations of olanzapine ■ Smoking increases the olanzapine clearance by 40% ■ In females, olanzapine clearance is decreased by 30% ■ Moderate risk for QTc prolongation	[S13, S17, S60, S62, S82, S84, S85, S94, S95]
	Quetiapine [N05AH04]	Anticholinergic hypothesis	CYP3A4 substrate	PO formulation reaches a peak plasma concentration 1.5 h after administration and has a half‐life of 6 h Require dose adjustment in patients with hepatic dysfunction. Renal dose adjustment is not necessary	Temporal disorientation, visual hallucination	■ Adjunct for ventilated patients in ICU settings	[S6, S18, S60, S84, S85, S95]
	Risperidone [N05AX08]	Has a higher affinity for 5‐HT2A receptors compared to D2, and reduces dopaminergic and serotonergic activity	CYP2D6 substrate	Risperidone reaches peak serum concentrations in 1–2 h	Extreme excitement hallucination, neuroleptic malignant syndrome	■ Long‐acting injection increases risk ■ CYP2D6 inhibitors like fluoxetine and paroxetine increase the plasma concentrations of risperidone ■ Risperidone can cause delirium compared to placebo (95% CI, 0.09–0.86; *p* = 0.02) ■ Compared to placebo, patients had more extrapyramidal effects (95% CI, 0.09–1.37; *p* = 0.03)	[S2, S4, S13, S17, S60, S66, S71, S84, S95]
	Lorazepam [N05AD01]	Potent D2 blocker	CYP3A4 substrate CYP2D6 inhibitor CYP1A2 substrate	2–30 mg/day	Agitation, perceptual disturbance, sleep–wake cycle disruption	■ Treatment of delirium for max. 1 week or less ■ Duration of action may be increased substantially in the setting of systemic ■ Inflammatory states (e.g., sepsis)	[S13, S18, S31, S46, S60, S79, S98, S104]
Anxiolytics/Hypnotics	General information	—	—	Daily equivalent dose of 5 mg or more statistically significantly increased the risk as did cumulative daily doses of benzodiazepines above 2 mg	Delusion	■ Long‐acting agents trended toward a stronger association compared to short‐acting benzodiazepines ■ Benzodiazepine withdrawal	[S3, S5, S17, S33, S53, S105]
	Midazolam [N05CD08]	Binds to GABA receptors, thereby inhibiting dopamine release	CYP3A4 substrate	Daily dose of 5 mg to a patient who is both coma‐ and delirium‐free will increase the odds by 4%	Delirium	■ Delirium‐inducing drug commonly used in palliative medicine	[S13, S46, S104]
	Zopiclone [N05CF01]	It being highly plasma‐protein bound (92.5%), its high drug–drug interaction risk by means of hepatic cytochrome P450‐3A4	CYP3A4	Single dose of 7.5 mg zopiclone	Disorientation agitation, hallucination, speech disturbance	■ Zolpidem plasma concentration is gender based	[S11, S47]
Antidepressant	General information	Anticholinergic action and tetracyclic antidepressant are α‐2 adrenergic receptor antagonists	—	—	—	■ Delirium inducing drugs commonly used in palliative medicine ■ Antidepressants had a disproportional association with delusion as a symptom	[S17, S28, S41, S46, S64]
	Duloxetine [N06AX21]	Causes dopaminergic excess	CYP2D6 inhibitor	N/A	Visual hallucination, sleep alteration, delusion	■ N/A	[S5, S63, S92]
	Amitriptyline [N06AA09]	Anticholinergic	CYP2D6 substrate	Overdose 1000 mg/day	Confusion	■ All tricyclic antidepressant drugs exert an anticholinergic effect, with amitriptyline having the strongest and nortriptyline the weakest	[S31, S46, S77]
	Bupropion [N06AX12]	Dopamine reuptake inhibition Possible inhibition of CYP2D6 may increase the concentration of hydroxybupropion and cause further dopaminergic excess and subsequent delirium	CYP2D6 inhibitor		Disorientation auditory and visual hallucination, persecutory delusion	■ Lower rates of sexual dysfunction than escitalopram, fluoxetine, paroxetine, and sertraline ■ Its use is not commonly associated with the side effect of dopamine excess ■ Bupropion‐related delirium is rare	[S63, S105]
Corticosteroid	General information	Steroid induced sleep disruption	CYP3A4 inducers (weak)	High‐dose pulse steroids cumulative daily doses of corticosteroids above 15 mg	Acute hyperactive delirium with psychosis, delusion, confusion	■ Intra‐articular administration	[S13, S51, S58, S68, S72, S77, S78]
Cardiovascular	General information	—	—	—	Cognitive impairment, simple acute confusion, visual hallucination	■ CV drugs with central nervous system (CNS) bioavailability carry the highest risk (disopyramide, quinidine, digoxin, clonidine, methyldopa, propranolol, reserpine) ■ Delirium was more common with calcium channel blockers than with renin–angiotensin system agents (∼40% higher) but less common than with beta‐blockers (∼20% lower) ■ Visual hallucinations have been reported with angiotensin‐converting enzyme (ACE) inhibitors in older patients, particularly those with a history of dementia or mild cognitive impairment	[S4, S13, S36, S38, S49, S67]
	Digoxin [C01AA04]	Hypokalemia and hypomagnesemia due to thiazide and loop diuretics may increase digoxin toxicity.	Not metab. by CYP	—	Visual hallucination, bilateral ballism	—	[S19, S42, S67]
	Clonidine [C02AC01]	Presynaptic alpha2‐receptor agonist inhibiting the release of norepinephrine.	CYP2D6 substrate	Risk is increased when medications are used in high or toxic doses	Confusion, lapses in memory, poor concentration	■ Clonidine can cause disruption in the release of various catecholamines and/or serotonin (5‐HT) in cortical and subcortical regions	[S13, S22, S67]
	Reserpine [C02AA02]	Reserpine depletes stores of 5‐HT, dopamine, and norepinephrine in the CNS	Metab. by the CYP system to a major degree but neither induces nor inhibits CYP activity	Risk is increased when medications are used in high or toxic doses	Confusion, lapses in memory, poor concentration	■ Relative lack of hepatotoxicity	[S67]
Antibiotics	General information	—	—	—	Delusion	■ No evidence that any particular antibiotic or class of antibiotics has a consistent or predictable adverse effect on cognition ■ Quinolone and Macrolide carry the greatest risk ■ Fluoroquinolones are a rare cause of seizures and delirium	[S4, S5, S11, S13]
	Tigecycline [J01AA12]	Glutamate receptor activation A substrate for p‐glycoprotein (P‐gp)	Not metab. via CYP	Infusion with a loading dose (mg not specified)	Delirium	■ Delirium as a side effect of tigecycline is very rare ■ As it does not metabolize via CYP, pharmacokinetic drug interactions are uncommon	[S66, S106]
	Ciprofloxacin [J01MA02; S01AE03; S02AA15; S03AA07]	N/A	CYP1A2 inhibitor	N/A	Mania, Insomnia, psychosis	■ The number of reports of delirium is greater for quinolone and macrolide antibiotics ■ Switchen to a cephalosporine	[S1, S11, S13, S46]
	Moxifloxacin [J01MA14/ S01AE07]	N/A	Not metab. via CYP	N/A	Confusion, attention alteration, temporal and spatial disorientation	■ The number of reports of delirium is greater for quinolone and macrolide antibiotics ■ Switchen to a cephalosporine	[S5, S80]
	Gatifloxacin [J01MA16; S01AE06]	N/A	Negligible inhibitors of CYP1A2 and CYP2C9	400 mg	Delusion, concern, agitation	■ Delirium as a side effect of gatifloxacin is rare	[S87]
	Scopolamine [A04AD01; N05CM05; S01FA02]	Competitive inhibition of antimuscarinic receptors	CYP3A4	11.7 (3–27) h after the application of the patch. The mean dosage of scopolamine was 2.25 mg	Insomnia, restlessness, disorientation, visual hallucination, repetitive behavior, gait disturbance, dysarthria, delusion	■ Transdermal application increases the risk	[S4, S13, S88]

**TABLE 4:** Risk associated with opioid analgesics in older patients with and without dementia.

Drug	Opioid characteristics and delirium‐specific risk	Recommendations in dementia
Similar risk of delirium in opioid‐naive patients among morphine, oxycodone, and tapentadol oral opioids.		
Buprenorphine patches	Patients potentially experience fewer side effects compared to other opioids.	■ Could be a good choice in dementia but reduced elimination due to its lipophilic character can result in increased drug plasma concentration in older patients.
Codeine	Considerable variation in response and adverse effects can increase the risk of falls.	■ Poorly tolerated in dementia.
Dextropropoxyphene	Has a propensity for neural and cardiac toxicity.	■ Best avoided in dementia.
Fentanyl patches	Their half‐life of 6–17 h makes treating side effects more difficult. Even after patch is removed, it will take at least 24 h for a significant drop in plasma concentration.	■ Useful in chronic pain and palliative care, although it should not be used to initiate opioid analgesia in frail older people. ■ The incidence of delirium after the commencement of fentanyl injection was significantly lower. ■ Transdermal fentanyl can be switched to slow‐release morphine (260 mg/day), allowing pain control, but the risk of hallucinations and somnolence remains high.
Methadone	Methadone has a very long half‐life (8—59 h), with an average of 24 h. Methadone has no ceiling effect.	■ Methadone is best avoided in dementia.
Morphine	Active metabolites penetrate the blood–brain barrier and are renally excreted, so they may accumulate in renal impairment, making older and frail patients more susceptible to adverse effects.	■ Very effective analgesic but likely to cause cognitive problems and other adverse effects in older patients. ■ Due to the blood–brain barrier penetration, slow‐release morphine increases the risk of delirium in dementia with Lewy bodies.
Oxycodone	Has a short half‐life (*t*_1/2_ = 3 h). Therefore, oxycodone has fewer drug–drug interactions and a more predictable dose–response relationship compared to other opiates. Oxycodone is associated with a lower risk of delirium.	■ Due to its more predictable dose–response relationships, oxycodone is a good candidate for oral analgesia in dementia patients.
Pentazocine	Risk of neurotoxicity.	■ Best avoided.
Pethidine	Pethidine was associated with a higher risk of causing delirium than oxycodone, as anticholinergic metabolites accumulate rapidly in renal impairment.	■ Pethidine carries a particularly high risk of cognitive impairment in patients with dementia.
Tramadol	High risk of drug interactions, considerable variation in response, and adverse effects may induce seizures. Tramadol IM should only be used after careful consideration in older adults.	■ May be poorly tolerated in dementia. ■ Avoid in patients with epilepsy or on other epileptogenic drugs, for example, psychotropics and memantine.

Note: Adapted from [S11].

#### 3.2.2 Antipsychotics

Typical and atypical antipsychotics are a significant risk factor for delirium, particularly those with anticholinergic effects [S46] such as clozapine [S84], olanzapine [S94], and levomepromazine [S46, S57]. While some reports suggest that haloperidol carries an increased risk of inducing a persistent delirium (OR 2.88; 95% CI, 1.38–6.02), particularly when administered intravenously [S2, S79, S82]. However, a meta‐analysis and GRADE‐level evidence ratings could not show an increased delirium risk for haloperidol (OR 0.96; 95% CI, 0.72–1.28) [S82]. Quetiapine is known to cause increased agitation, temporal disorientation, and visual hallucinations [S6]. Quetiapine is often used as a sedation adjunct for ventilated patients in the ICU setting with a *t*_1/2_ of 6 h [S84, S85]. Despite reports about their significant potential in the causation of delirium, the majority of reported information relates to their off‐label use in the prevention and management of delirium. The effectiveness of antipsychotics in the prevention and management of delirium and its associated symptoms is, however, questionable as a meta‐analysis found that antipsychotic use was not associated with changes in the duration or severity of delirium [S14, S16]. In addition, QTc prolongation is a common side effect for many antipsychotics, with IV haloperidol significantly increasing the risk of arrhythmias and other cardiovascular concerns [S84]. This is reported to be less of a problem with atypical antipsychotics [S84].

#### 3.2.3 Anxiolytics/Hypnotics

Long‐acting benzodiazepines are associated with a higher risk of delirium compared to short‐acting ones [S4, S13, S17, S19, S28, S33, S43, S60]. Cumulative daily doses of benzodiazepines above 2 mg (HR, 2.04; 95% CI, 1.05–3.97) and 5 mg (OR 3.5; 95% CI, 1.4–8.8) have been reported to carry a delirium risk [S11, S34]. Benzodiazepine withdrawal has also been reported to be a risk factor [S105]. While the retrieved literature does not describe the mechanism by which benzodiazepines cause delirium, Z‐substances (zolpidem, zopiclone, zaleplon, and eszopiclone) risk seems to describe a disruption in the dopaminergic and GABAergic systems, with single doses of 5 mg zolpidem or 7.5 mg zopiclone having been linked to symptoms such as visual hallucinations, disorientation, and agitation [S47].

#### 3.2.4 Antidepressants

Tricyclic antidepressants (TCAs) and selective serotonin reuptake inhibitors (SSRIs) in particular are reported to be associated with an increased risk of developing delirium and akathisia [S46]. Paroxetine is the SSRI reported to carry the highest risk [S17, S4], while amitriptyline is the most frequently mentioned TCA in this SR [S5, S15, S18, S46, S76, S77].

#### 3.2.5 Corticosteroids

While all steroids are considered to carry a risk of delirium [S4, S13, S17, S19, S33, S34, S36, S46, S51, S54, S58, S72, S77], patients exposed to cumulative daily doses of corticosteroids above 15 mg (HR, 2.67; 95% CI, 1.18–6.03) presented an increased risk of developing delirium within 28 days of hospitalization according to a study of 261 hospitalized cancer patients [S34].

#### 3.2.6 Cardiovascular

Especially cardiovascular (CV) drugs with central nervous system (CNS) bioavailability carry the highest risk of acute confusion and delirium, which may even result in more chronic changes in cognition (disopyramide, quinidine, digoxin, clonidine, methyldopa, propranolol, reserpine) [S4, S13, S22, S49, S67]. Digoxin, due to its neurotoxic effects and its risk of causing hypokalemia and hypomagnesemia, especially in combination with thiazide and loop diuretics, is most frequently mentioned in association with delirium [S4, S11, S17, S43, S67]. However, no specific mechanisms, dosing instructions, or treatment alternatives are reported. While calcium channel blockers have been reported to be associated with a higher rate of delirium than renin–angiotensin system agents (∼40% higher) but less common than in beta blockers (20% lower), no specific information about dosing, and so forth is provided beyond the generic statement [S38].

#### 3.2.7 Cholinesterase Inhibitors

Cholinesterase inhibitors can cause delirium due to their effects on the cholinergic system, especially in individuals with pre‐existing cholinergic system disruptions or high anticholinergic burden [S28]. Even CNS‐active drugs that are appropriately used in this population can accumulate in amounts that lead to problems such as delirium, sedation, and falls. Symptoms most commonly reported include agitation and hallucinations [S21, S28, S29, S50]. The response to these drugs can vary significantly among individuals, leading to inconsistent outcomes regarding delirium [S1, S4, S17, S69, S76, S83, S85, S106].

#### 3.2.8 Antibiotics

Reports on antibiotics in association with delirium, while frequent, are very disparate in nature. There seems to be no conclusive evidence that any particular antibiotic or class of antibiotics has a consistent or predictable adverse effect on cognition. However, most antibiotics may occasionally be associated with delirious‐like episodes [S4, S5, S11, S27, S37, S46, S48, S49, S80, S82, S87, S90, S13]. This is also true for the reported symptoms, which seem to range from general delusions across a wide spectrum to insomnia, restlessness, disorientation, visual hallucination, repetitive behavior, gait disturbance, dysarthria, and delusion [S4, S11]. It is stated that the number of reports of delirium is greatest for quinolone and macrolide antibiotics [S11], results that were corroborated by a recent pharmacovigilance analysis of 4559 reports of delusion [S5].

### 3.3 Medication Combinations

Medication combinations that carry a high risk of inducing a delirium are shown in Table S5. This heat map depicts the frequency of reporting across all included studies [S5, S18, S49, S91]. In a retrospective case‐control study including 28,503 individuals (> 65 years) with delirium, published in 2021, a time‐trend‐adjusted matched odds ratio (MOR) analysis identified several combinations that are significantly associated with delirium. Combined exposures to quetiapine and furosemide (MOR = 3.74; 95% CI, 1.74–8.00) and fentanyl exposure (MOR = 5.31; 95% CI, 2.52–11.20) within 3 days of exposure. Also, haloperidol exposure alone (MOR = 3.74; 95% CI, 2.60–5.41) and combined exposure to haloperidol, docusate, and paracetamol (MOR = 6.06; 95% CI, 2.91–12.63) within a 7‐day exposure showed a significant association with delirium [S18]. As where the combined exposures to furosemide, omeprazole, and lorazepam (MOR = 3.94; 95% CI, 3.03–5.10), and fentanyl (MOR = 3.46; 95% CI, 2.05–9.21). A retrospective prevalence study including 22,923 older patients (> 65 years) also identified the combinations of antidepressants + antihypertensives (*n* = 3354 [28.6%]), psycholeptics + antihypertensives (*n* = 2082 [17.75%]), and opioids + antihypertensives (*n* = 2084 [17.77%]) [S49]. Oxycodone + hydromorphone, apomorphine + carbidopa, and rotigotine + carbidopa were among the most frequently reported combinations in association with delusions in a pharmacovigilance analysis study of 4559 side effect reports [S5].

### 3.4 Treatment

While antipsychotic use is by far the most frequently used approach to pharmacological treatment of delirium, their use is controversial [S56]. Several SRs and meta‐analyses have failed to show an association between antipsychotic use and the duration of severity of delirium [S1, S17, S57]. Both the Beers Criteria and the STOPPFall risk tool recommend the withdrawal of antipsychotics in older adults unless they are at risk of significant harm to self and others [S57] (Table 5).

**TABLE 5:** Risk stratification of Antipsychotic medications used in management of delirium symptoms.

Medication	Route of administration	Typical dosing range	Considerations	Supporting information references
Ziprasidone	PO, IM, and IV	20–160 mg/day	Moderate D2 blockade Conflicting association with QTc prolongation	[S13, S84, S85]
Haloperidol	PO, IM, and IV	2–30 mg/day	Potent D2 blockade Minimal sedation FDA warning for QTc prolongation for the IV form Avoid oversedation Can increase extrapyramidal side effects May have a negative impact on overall survival	[S2, S4, S13, S17, S18, S31, S60, S65, S79 S82, S84, S89, S95]
Olanzapine	PO, IM, and IV	2.5–30 mg/day	Moderate D2 blockade Moderate sedation Metabolic side effects (weight gain, hyperglycemia) Reduced blood pressure *Effective for critically ill patients with delirium* Rare association with serotonin syndrome in combination with serotonergic antidepressants Its anticholinergic effects may worsen delirium (rare) CYP1A2 metabolized concentration and duration of action may be increased substantially in the setting of systemic inflammatory states (e.g., sepsis)	[S82, S85, S95]
Quetiapine	PO	12.5–1200 mg/day	Minimal D2 blockade Highly sedating Has been reported to induce delirium Off‐label for the management of delirium and as a sedation *adjunct for ventilated patients in the ICU setting* Reaches a peak plasma concentration 1.5 h after administration and has a half‐life of 6 h. Requires dose adjustment in patients with liver disease	[S6, S32, S54, S85]
Risperidone	PO	0.5–16 mg/day	High D2 blockade Mildly sedating Oral formulation is available for the *management of aggressive or agitated behavior in adults with Alzheimer's disease* and other mental health conditions, including delirium Reaches peak serum concentrations in 1–2 h and is metabolized by the cytochrome CYP2D6 enzyme Risperidone causes a moderate degree of QTc prolongation Risperidone itself can cause delirium compared to placebo (95% CI, 0.09–0.86; *p* = 0.02)	[S2, S4, S13, S17, S60, S66, S71, S84, S95]
Chlorpromazine	PO, IM, and IV	200–800 mg/day	More sedating than high potency agents Reduced blood pressure	[S84]
Apiprazole			Lack of sedative property No significant QTc prolongation May be an option for *delirium patients who do not require substantial sedation* At least as efficient as haloperidol with a proposed better tolerability and safety profile	[S65, S84, S92]

Note: Adapted from [S84].

Dexmedetomidine has the potential to improve delirium outcomes [S14, S16, S24, S85, S99], while orexin antagonists suvorexant and lemborexant may prevent delirium in patients with a wide range of medical conditions [S39, S40].

### 3.5 Treatment Alternatives

There is a distinct paucity of specific information about therapeutic alternatives for medication causing delirium. Strategies center mainly on discontinuation of medication, being mindful of the anticholinergic potential of individual medication, and choosing a newer generation of medication within a medication class [S4, S11, S12, S45, S57]. Especially, the potential for anticholinergic intoxication is mentioned, with one paper suggesting that physostigmine, a nonselective acetyl‐cholinesterase inhibitor, could be used for anticholinergic intoxication [S30, S64, S76].

### 3.6 Medication Associated With Delirium in Dementia Patients

According to a study dating back to 2004, prescribed medication is the cause of delirium in between 12% and 39% of dementia cases [S11]. Mechanisms are usually associated with one of the four neurotransmitters involved in cognition: acetylcholine, histamine, gamma‐aminobutyric acid (GABA), and opioid peptides [S11]. Consequently, the drug classes most often mentioned in this review across all included papers are cholinesterase inhibitors (*n* = 6 [5.6%]), antibiotics (*n* = 3 [2.8%]), analgesics (*n* = 3 [2.8%]), cardiac drugs (*n* = 2 [1.8%]), antimanika (*n* = 2 [1.8%]), and analgesics (Figure 2). In particular, memantine and donepezil (> 5 mg/day) have been associated with an increased risk of DSD [S68–S70, S76, S83, S102], where various dementia subtypes were mentioned with no obvious reporting patterns. Visual hallucinations have also been reported with rivastigmine transdermal treatment [S21], while galantamine is mentioned, but no further detail is given [S29]. Analgesics formed an exception, with the opioid‐associated risk in patients with and without dementia being summarized in Table 4 [S11]. Oxycodone has the most predictable dose–response relationship due to its short half‐life (*t*_1/2_ = 3 h), resulting in a lower risk of delirium and a faster washout period [S11].

## 4 Discussion

Overall, there is a paucity of specific and good‐quality medication‐related publications associated with the medication risks in relation to delirium, its mechanisms, treatment, and treatment alternatives in adult patients with and without dementia. Neurotransmitter imbalances, pharmacokinetic changes, and physiological processes are frequently reported as medication‐related delirium‐causing mechanisms. A total of *n* = 158 individual medications were listed across 20 different drug classes, with the riskiest drug combinations being the multiple use of opioids and dopamine agonists. Despite good‐quality evidence from several large real‐world data studies, a large disparity in the reporting of risky combinations was identified. No evidence for the use of antipsychotics in the treatment of delirium could be reported. Alternative pharmacological strategies focus on the mindful use of anticholinergic medication and choosing a newer generation of medication. Medication‐related disruption of acetylcholine, histamine, and GABA is associated with delirium in patients with an underlying dementia diagnosis.

The inherent lack of understanding of medication risk in relation to either association or causation, as well as treatment of delirium in adult patients with and without dementia, is reflective of the general lack of understanding of the pathophysiology of delirium irrespective of dementia diagnosis (Wilson et al. 2020; Gupta et al. 2008; Iglseder et al. 2010). This fundamental gap in our understanding is particularly important for patients taking multiple medications, as they are often multimorbid and frail, making them especially vulnerable to delirium. While some mechanisms are easily explained such as the strong association of opioids, in combination or alone, with delirium due to the neurodegenerative properties of mitochondrial dysfunction, oxidative stress, and the upregulation of apoptotic proteins, mitochondrial dysfunction itself has been shown to be involved in the pathophysiology of delirium (Guo et al. 2013; Liu et al. 2022; Lu et al. 2020; Gunther et al. 2008). Other mechanisms, such as anticholinergic medication and their association with delirium, are less well understood while the association as a significant risk factor is well documented, the exact mechanisms of neurotransmitter disturbance caused by the different anticholinergic drugs or drug combinations require further investigation (Talabaki et al. 2024; Macha et al. 2023). Interestingly, rivastigmine, an acetylcholinesterase inhibitor, has been identified as a potential treatment for anticholinergic delirium, despite other reports implicating cholinesterase inhibitors in its development (Fratta et al. 2023; Schlake et al. 2024). While the evidence summarized from the papers included in this review did not identify cholinesterase inhibitors as a treatment option, it did seem to implicate this drug group in the development of delirium symptoms, especially in dementia patients [S5, S28, S29, S50, S62, S68–S70, S76, S83, S90, S102]. Specific information on medication risks associated with DSD was sparse. This reflects the only two SRs to date aiming to summarize the published information on prevalence, associated features, outcomes, and management of DSD, despite it having a pooled prevalence estimated to be as high as 48.9% in older hospitalized patients (Fick et al. 2002; Han et al. 2022). The SR by Han et al. (2022) only lists psychotropic medication, antibiotics, and polypharmacy as the medication risk factors for DSD without providing any medication‐related detail. Our study was able to provide more detailed medication‐related information, identifying cholinesterase inhibitors, antibiotics, analgesics, cardiac drugs, antimanika, and analgesics as carrying an increased delirium risk in patients with dementia (Figure 1), in particular, memantine and donepezil (> 5 mg/day). Among antibiotics, nitrofurantoin, gatifloxacin, clarithromycin, and macrolides were associated with an increased risk of DSD, while detailed opioid analgesic prescribing advice in older patients with and without dementia was summarized (Table 4). This information potentially provides the most comprehensive information on medication‐associated delirium risk in dementia patients to date.

Among the lack of insight into complex medication‐related risk factors, the search for obvious patterns and unifying mechanisms continues. Considering the drug classes associated with delirium, their varying effect on the vagus nerve seems to offer one such possible explanation. The vagus nerve is key in the transmission of signals between the gastrointestinal tract and the central nervous system, the gut–brain axis (GBA) (Bonaz et al. 2018). Both the pathophysiology of cognitive impairment and dementia have been linked to the vagus nerve and GBA (Ticinesi et al. 2018; Zhang et al. 2022). Medications, such as antibiotics, corticosteroids, and proton pump inhibitors, have been reported to significantly alter the gut microbiome, impacting both its composition and function and leading to a profound effect on health, including drug efficacy and toxicity (Barrio et al. 2022; Zhao et al. 2023; Simpson et al. 2021). Especially, medication such as antibiotics, corticosteroids, and proton pump inhibitors significantly impact the gut microbiome (Fossmark and Olaisen 2024; Couch et al. 2023). A recent study investigating whether microbiome‐targeted therapies (MTTs) improve cognition and prevent postoperative cognitive dysfunctions such as delirium reported that MTTs decrease inflammation and have a positive effect on cognition. While this line of research is still in its infancy, the SR concludes that MTTs could be a potential new preventative strategy for cognitive impairment after surgery (Sugita et al. 2023; Garcez et al. 2023). A study investigating the association between gut microbiota and delirium occurrence in acutely ill older adults identified that gut microbiome diversity and composition were significantly different in acutely ill hospitalized older adults who experienced delirium (Garcez et al. 2023).

As long as “delirium” is used as a blanket term for a plethora of symptoms and etiological heterogeneity without its categorization into distinct etiological subgroups, it will be very difficult to design high‐quality studies that help to develop clear prescribing advice and delirium interventions (Ormseth et al. 2023; Oh et al. 2020). However, it is evident that no single pharmacological intervention will demonstrate effectiveness, such a comprehensive guideline can help to support multicomponent approaches such as the Hospital Elder Life Program (HELP), which have demonstrated to reduce the odds of delirium by > 50% in the intervention group compared to controls (OR 0.47; 95% CI, 0.37–0.59; *I* ^2^ = 28%; *n* = 3605 patients) (Hshieh et al. 2018). In the meantime, the increased use of artificial intelligence in healthcare poses additional opportunities for delirium care (Gong et al. 2023; Xie et al. 2022). Very little information however is available on subsequent treatment and pharmacological intervention strategies much less how AI can help with identifying underlying medication mechanisms and treatment strategies (Weidmann and Watson 2024).

## 5 Implications for Practice

This is the only review to provide a comprehensive summary of medication‐related information in relation to delirium and dementia, which can be used to support prescribing decision‐making in adult patients and can help to support multicomponent approaches to delirium care. In addition to the heterogeneity of reported underlying mechanisms of action of common medications associated with delirium, it discusses a possible association between the effect of medication on the gut microbiome and delirium. Results presented in this SR support the call for categorizing delirium into distinct etiological subgroups to facilitate the design of high‐quality studies that help to distinguish contributing medication factors and to develop clear prescribing advice and delirium interventions.

Papers specifically looking at delirium superimposed on dementia (DSD) were not included in the search strategy resulting in incomplete information presented. The research team registerred andconducted a separate Systematic Review focused specifically on delirium superimposed on dementia (DSD) [PROSPERO: CRD42024546118] [Aslam et al. 2024].

## 6 Future Research

Given the plethora and heterogeneity of identified contributing factors that contribute to the development of delirium according to Ormseth et al. (2023), it seems unlikely to adequately address the paucity of good‐quality studies that address all these factors. In relation to the role of medication in delirium, future studies should focus on the development of a comprehensive guideline and electronic prescribing resource summarizing detailed pharmacological information on the causation, treatment, and prevention of delirium in different patient groups to support existing and future multicomponent treatment approaches and support prescribing practice and patient care. Further, the use of artificial intelligence to support clinical pharmacy and clinical pharmacology research in the identification and development of new and novel therapeutic approaches to delirium in different patient groups should be explored. Finally, the association between the impact of medications on the GBA and gut microbiome along with their associated risk in the causation, prevention, and treatment of delirium requires further investigation.

## 7 Conclusion

There is still an inherent lack of understanding of medication risk associated with the causation and treatment of delirium in adult patients with and without dementia, which is reflective of the general lack of our understanding of the pathophysiology of delirium. While it is not clear if medication is a contributing or precipitating factor for delirium, this review has identified many more drug classes and individual drugs than are typically listed in standard practice guidance. The reported effect of medication on the gut microbiome and vagus nerve may pose a unifying mechanism for medication involvement in delirium, especially in patients whose health is already extremely challenged. Clinical pharmacists, as experts in medicine, should always be included in multicomponent approaches to the treatment of delirium.

## Author Contributions

**Anita Elaine Weidmann**: conceptualization, data curation, formal analysis, methodology, project administration, supervision, validation, writing – original draft, writing – review and editing. **Rut Matthíasdóttir**: data curation, formal analysis, writing – review and editing. **Guðný Björk Proppé**: data curation, validation, writing – review and editing **Ivana Tadić**: formal analysis, writing – review and editing. **Pétur Sigurdur Gunnarsson**: conceptualization, methodology, writing – review and editing. **Freyja Jónsdóttir**: conceptualization, formal analysis, methodology, project administration, supervision, validation, writing – review and editing.

## Peer Review

The peer review history for this article is available at https://publons.com/publon/10.1002/brb3.70706.

## Supporting information




**Supplementary Table S1**.: Systematic Review inclusion criteria as detailed on the PROSPERO registered protocol [CRD42022366020]
**Supplementary Table S2**.: Systematic Database search results

## Data Availability

All data generated or analyzed during this study are included in this published article and its supplementary information files.
