# Supplementary material for: Medication Causes and Treatment of Delirium in Patients With and Without Dementia
Source: Brain Behav. 2025 Jul 21;15(7):e70706. doi: 10.1002/brb3.70706 (PMC12277658; doi:10.1002/brb3.70706)
Supplement: Supplementary file 1 — Supplementary Table S1.: Systematic Review inclusion criteria as detailed on the PROSPERO registered protocol [CRD42022366020] Supplementary Table S2.: Systematic Database search results [file BRB3-15-e70706-s001.docx]

**Supplementary files**

***Supplementary Table S1.:*** *Systematic Review inclusion criteria as detailed on the PROSPERO registered protocol* [CRD42022366020]

| Inclusion criteria |
| --- |
| - Original, peer-reviewed studies, systematic and narrative reviews, case reports, commentaries, editorials and scientific communication (e.g. conference abstracts) - Documents detailing the management of medication induced delirium. - Documents detailing medication induced delirium mechanisms and therapeutic alternatives. - Delirium medication in the context of dementia. - All clinical settings except for the peri-operative setting. - Documents published since 2000. - No geographical exclusions - Language English & Icelandic |

**Supplementary Table S2.:** *Systematic database search results*

| Date | Database | Keywords | Hits |
| --- | --- | --- | --- |
| 03.09.2024 | PubMed | ((((("Delirium"[Mesh]) OR (delirium)) OR (hallucinations)) OR ("Hallucinations"[Mesh])) AND (((drug induced) OR ("Drug-Related Side Effects and Adverse Reactions"[Mesh])) OR ("chemically induced" [Subheading]))) AND (("Dementia"[Mesh]) OR (dementia)) | 397 |
| 31.08.2024 | EBSCO-CINAHL | ( delirium OR hallucinations ) AND ( "drug induced" OR "Drug-Related Side Effects and Adverse Reactions" ) AND dementia | 17 |
| 31.08.2024 | EBSCO-IPA | ( delirium OR hallucinations ) AND ( "drug induced" OR "Drug-Related Side Effects and Adverse Reactions" ) AND dementia | 7 |
| 31.08.2024 | EBSCO-APA PsycArticles | ( delirium OR hallucinations ) AND ( "drug induced" OR "Drug-Related Side Effects and Adverse Reactions" ) AND dementia | 11 |
| 31.08.2024 | Cochrane library | ((((("Delirium"[Mesh]) OR (delirium)) OR (hallucinations)) OR ("Hallucinations"[Mesh])) AND ((drug induced) OR ("Drug-Related Side Effects and Adverse Reactions"[Mesh])) AND (("Dementia"[Mesh]) OR (dementia)) | 13 |
| 03.09.2024 | SAGE Journals | (delirium OR hallucinations ) AND ( "drug induced" OR "Drug-Related Side Effects and Adverse Reactions" ) AND dementia | 580 |
| 03.09.2024 | Science Direct | (delirium OR hallucinations) AND ("drug induced" OR "Drug-Related Side Effects and Adverse Reactions"[Mesh]) AND ("dementia"[Mesh] OR dementia) | 714 |
| 31.08.2024 | SCOPUS | TITLE-ABS-KEY (( "Delirium" [mesh] ) OR ( "delirium" ) OR ( "hallucinations" ) OR ( "Hallucinations" [mesh] ) ) AND TITLE-ABS-KEY ( "drug induced" OR ( "Drug-Related Side Effects and Adverse Reactions" [mesh] ) ) AND TITLE-ABS-KEY ( ( "Dementia" [mesh] ) OR ( "dementia" ) ) ) | 269 |
| 31.08.2024 | Web of Science Core Collection | ((ALL=(delirium OR hallucination)) AND ALL=("drug induced" OR "Drug-Related Side Effects and Adverse Reactions"[Mesh])) AND ALL=(dementia) | 152 |
| 31.08.2024 | National Grey Literature Collection | (Delirium OR hallucinations) AND (“drug induced” OR "Drug-Related Side  Effects and Adverse Reactions”) AND (dementia) | 3 |
| 31.08.2024 | Google scholar-grey literature - Ovid | ( delirium OR hallucinations ) AND ( "drug induced" OR "Drug-Related Side  Effects and Adverse Reactions" ) AND dementia | 1690 |
| 03.09.2024 | Open Access theses and Dissertations | (Delirium OR hallucinations) AND (“drug induced”) AND (dementia) | 14 |
| Total |  | **N = 3867** | |

**Identification of studies via other methods**

**Identification of studies via databases and registers**

Records removed *before screening*:

Duplicate records removed

**n = 658**

Studies conducted prior to 2000 **n=637**

Records identified from scientific publication database searching:

(PubMed n=397; CINAHL n=17; IPA n=7; PSychArticles n=11; Cochrane library n=13; SAGE n=580; Science Direct n=714; SCOPUS n=269; Web of Science Core Col. n=152; Nat, Grey Lit. n=3; Open Access theses and diss, n=14; Google Scholar n=1690)

**TOTAL n=3867**

**Identification**

Independent Title screening

**n = 2572**

Titles excluded

**n = 1535**

Additional record through reference list searching and conference abstracts **n = 20**

Independent Abstract screening

**n = 1037**

Abstracts excluded

**n = 679**

**Screening**

Independent Full text screening

**n = 378**

Full texts excluded:

Full text not available n=62

Not drug-induced delirium n=81

Peri-op. delirium n = 9

Treatment n = 11

Underage n = 1

Before 2000 n = 11

Language n = 1

Not relevant n = 96

**TOTAL n = 272**

Studies included in review

**n = 106**

***Supplementary Figure S1.:*** *PRISMA flowchart showing the identification, screening and selection of delirium publications across 12 scientific publication databases.*

**Included**

**Supplementary Table S3.:** *Systematic review references (n=106)*

| *Full-text Publications included in this SR* |
| --- |
| 1. Agar M, Lawlor P. Delirium in cancer patients: a focus on treatment-induced psychopathology. *Curr Opin Oncol*. 2008;20(4):360-6. 2. Agar MR, Lawlor PG, Quinn S, Draper B, Caplan GA, Rowett D, Sanderson C, Hardy J, Le B, Eckermann S, McCaffrey N, Devilee L, Fazekas B, Hill M, Currow DC. Efficacy of Oral Risperidone, Haloperidol, or Placebo for Symptoms of Delirium Among Patients in Palliative Care: A Randomized Clinical Trial. *JAMA Intern Med*. 2017;177(1):34-42. 3. Ahmed S, Leurent B, Sampson EL. Risk factors for incident delirium among older people in acute hospital medical units: a systematic review and meta-analysis. *Age Ageing*. 2014;43(3):326-33. 4. Alagiakrishnan K, Wiens CA. An approach to drug induced delirium in the elderly. *Postgrad Med J*. 2004;80(945):388-93. 5. Alexander Balcerac, Antoine Baldacci, Alix Romier, Sophie Annette, Baptiste Lemarchand, Kevin Bihan, Hugo Bottemanne, Drug-induced delusion: A comprehensive overview of the WHO pharmacovigilance database. *Psychiatry Research*. 2023; 327: 115365. 6. Almeida F, Albuquerque E, Murta I. Delirium Induced by Quetiapine and the Potential Role of Norquetiapine. *Front Neurosci*. 2019;13:886. 7. Anderson D. Preventing delirium in older people. *Br Med Bull*. 2005;73-74:25-34. 8. Arts MHL, Petrykiv S, Jonge L. 2019  Arts, M. H. L., Petrykiv, S., & de Jonge, L. Tramadol-induced delirium. *European Psychiatry*. 2019;56:S358-S359. 9. Bandyopadhyay A, Yaddanapudi LN, Saini V, Sahni N, Grover S, Puri S, Ashok V. Efficacy of melatonin in decreasing the incidence of delirium in critically ill adults: a randomized controlled trial. Crit Care Sci. 2024;36:e20240144en. 10. Benítez del Rosario MA, Feria M, Montón Alvarez FI. Hallucinations in an elderly cancer patient: opioid neurotoxicity or dementia with Lewy bodies? *Palliat Med*. 2002;16(1):71-2. 11. Bishara D, Harwood D. Safe prescribing of physical health medication in patients with dementia. *Int J Geriatr Psychiatry*. 2014;29(12):1230-41. 12. Bishara D. Anticholinergic action is rarely a good thing. *Therapeutic Advances in Psychopharmacology*. 2023;13. 13. Boyle DA. Delirium in older adults with cancer: implications for practice and research. *Oncol Nurs Forum*. 2006;33(1):61-78. 14. Burry LD, Cheng W, Williamson DR, Adhikari NK, Egerod I, Kanji S, Martin CM, Hutton B, Rose L. Pharmacological and non-pharmacological interventions to prevent delirium in critically ill patients: a systematic review and network meta-analysis. *Intensive Care Med*. 2021;47(9):943-960. 15. Cancelli I, Beltrame M, Gigli GL, Valente M. Drugs with anticholinergic properties: cognitive and neuropsychiatric side-effects in elderly patients. *Neurol Sci*. 2009;30(2):87-92. 16. Carayannopoulos, Kallirroi Laiya; Alshamsi, Fayez; Chaudhuri, Dipayan; Spatafora, Laura; Piticaru, Joshua; Campbell, Kaitryn; Alhazzani, Waleed; Lewis, Kimberley. Antipsychotics in the Treatment of Delirium in Critically Ill Patients: A Systematic Review and Meta-Analysis of Randomized Controlled Trials*. Critical Care Medicine. 2024; 52(7):p 1087-1096. 17. Catic AG. Identification and management of in-hospital drug-induced delirium in older patients. *Drugs Aging*. 2011;28(9):737-48. 18. Chyou TY, Nishtala PS. Identifying frequent drug combinations associated with delirium in older adults: Application of association rules method to a case-time-control design. *Pharmacoepidemiol Drug Saf*. 2021; 30:1402-1410. 19. Clegg A, Young JB. Which medications to avoid in people at risk of delirium: a systematic review. *Age Ageing*. 2011;40(1):23-9. 20. Cuigniez M, Audenaert K, Santens P, Heylens G. SILENT: The syndrome of irreversible lithium-effectuated neurotoxicity: A case report with two years follow-up. Clin Neurol *Neurosurg*. 2020;195:106057. 21. Değirmenci Y, Keçeci H. 2016  Visual Hallucinations Due to Rivastigmine Transdermal Patch Application in Alzheimer's Disease; The First Case Report. *International Journal of Gerontology*. 2016;10(4):240-1. 22. Dharmarajan TS, Dharmarajan L. Tolerability of Antihypertensive Medications in Older Adults. *Drugs Aging*. 2015;32(10):773-96. 23. Doane J, Stults B. Visual hallucinations related to angiotensin-converting enzyme inhibitor use: case reports and review*. J Clin Hypertens (Greenwich*). 2013;15(4):230-3. 24. Duprey MS, Dijkstra-Kersten SMA, Zaal IJ, Briesacher BA, Saczynski JS, Griffith JL, Devlin JW, Slooter AJC. Opioid Use Increases the Risk of Delirium in Critically Ill Adults Independently of Pain. *Am J Respir Crit Care Med*. 2021;204(5):566-572. 25. Dyer AH, Murphy C, Lawlor B, Kennelly SP; NILVAD StudyGroup. Sedative Load in Community-Dwelling Older Adults with Mild-Moderate Alzheimer's Disease: Longitudinal Relationships with Adverse Events, Delirium and Falls. *Drugs Aging*. 2020;37(11):829-837. 26. Edwards KR, O'Connor JT. Risk of delirium with concomitant use of tolterodine and acetylcholinesterase inhibitors. *J Am Geriatr Soc*. 2002;50(6):1165-6. 27. Feng Z, Huang J, Xu Y, Zhang M, Hu S. Dissociative disorder induced by clarithromycin combined with rabeprazole in a patient with gastritis. *J Int Med Res*. 2013;41(1):239-43. 28. Fick D, Kolanowski A, Waller J. High prevalence of central nervous system medications in community-dwelling older adults with dementia over a three-year period. *Aging Ment Health.* 2007;11(5):588-95. 29. Fisher AA, Davis MW. Prolonged QT interval, syncope, and delirium with galantamine. *Ann Pharmacother*. 2008;42(2):278-83. 30. Gale L, McGill K, Twaddell S, Whyte IM, Lewin TJ, Carter GL. Hospital-treated deliberate self-poisoning patients: Drug-induced delirium and clinical outcomes. *Aust N Z J Psychiatry*. 2022;56(2):154-163. 31. Gareri P, De Fazio P, Cotroneo A, Lacava R, Gallelli L, De Fazio S, De Sarro G. Anticholinergic drug-induced delirium in an elderly Alzheimer's dementia patient. *Arch Gerontol Geriatr.* 2007;44 Suppl 1:199-206. 32. Gareri P, Lacava R, Cotroneo A, Bambara V, Marigliano N, Castagna A, Costantino DS, Ruotolo G, de Sarro G. Valproate-induced delirium in a demented patient. *Arch Gerontol Geriatr.* 2009;49 Suppl 1:113-8. 33. Gaudreau JD, Gagnon P, Roy MA, Harel F, Tremblay A. Association between psychoactive medications and delirium in hospitalized patients: a critical review. *Psychosomatics*. 2005;46(4):302-16. 34. Gaudreau JD, Gagnon P, Harel F, Roy MA, Tremblay A. Psychoactive medications and risk of delirium in hospitalized cancer patients. *J Clin Oncol*. 2005;23(27):6712-8. 35. Gaudreau JD, Gagnon P, Roy MA, Harel F, Tremblay A. Opioid medications and longitudinal risk of delirium in hospitalized cancer patients. *Cancer*. 2007;109(11):2365-73. 36. Green AR, Reifler LM, Boyd CM, Weffald LA, Bayliss EA. Medication Profiles of Patients with Cognitive Impairment and High Anticholinergic Burden. *Drugs Aging*. 2018;35(3):223-232. 37. Han QYC, Rodrigues NG, Klainin-Yobas P, Haugan G, Wu XV. Prevalence, Risk Factors, and Impact of Delirium on Hospitalized Older Adults With Dementia: A Systematic Review and Meta-Analysis. *J Am Med Dir Assoc*. 2022;23(1):23-32.e27. 38. Harrison PJ, Luciano S, Colbourne L. Rates of delirium associated with calcium channel blockers compared to diuretics, renin-angiotensin system agents and beta-blockers: An electronic health records network study. *J Psychopharmacol*. 2020;34(8):848-855. 39. Hatta K, Kishi Y, Wada K, Takeuchi T, Taira T, Uemura K, Ogawa A, Takahashi K, Sato A, Shirakawa M, Herring WJ, Arano I; Suvorexant 085 Study Group. Suvorexant for Reduction of Delirium in Older Adults After Hospitalization: A Randomized Clinical Trial. JAMA Netw Open. 2024;7(8):e2427691. 40. Henmi R, Nakamura T, Mashimoto M, Takase F, Ozone M. Preventive Effects of Ramelteon, Suvorexant, and Lemborexant on Delirium in Hospitalized Patients With Physical Disease: A Retrospective Cohort Study*. J Clin Psychopharmacol*. 2024;44(4):369-377. 41. Herzig SJ, Anderson TS, Jung Y, Ngo LH, McCarthy EP. Risk factors for opioid-related adverse drug events among older adults after hospital discharge. *J Am Geriatr Soc*. 2022;70(1):228-234. 42. Holstein A, Hassan A, Patzer OM, Rohde M. Unusual neurological manifestation of severe digitoxin intoxication with bilateral ballism and visual hallucinations. *Aging Clin Exp Res*. 2015;27(3):391-3. 43. Hufschmidt A, Shabarin V, Zimmer T. Drug-induced confusional states: the usual suspects? *Acta Neurol Scand*. 2009;120(6):436-8. 44. Iqbal MM, Aneja A, Rahman A, Megna JL, Yasmin L, Schwartz TL, Osmany S, Alam MA. Therapeutic Options in the Treatment of Clozapine-Induced Adverse Effects.*J Pharm Tecnol*. 2004; 20(3):155-164. 45. Ito G, Kanemoto K. A case of topical opioid-induced delirium mistaken as behavioural and psychological symptoms of dementia in demented state. *Psychogeriatrics*. 2013;13(2):118-23. 46. Jackson N, Doherty J, Coulter S. Neuropsychiatric complications of commonly used palliative care drugs. *Postgrad Med J*. 2008;84(989):121-6; quiz 125. 47. Kalan U, Soysal P, Isik AT. Delirium associated with only one dose of zopiclone in an older adult. *Psychogeriatrics*. 2018;18(4):321-323. 48. Kass JS, Shandera WX. Nervous system effects of antituberculosis therapy. *CNS Drugs*. 2010;24(8):655-67. 49. Kassie GM, Kalisch Ellett LM, Nguyen TA, Roughead EE. Use of medicines that may precipitate delirium prior to hospitalisation in older Australians with delirium: An observational study. *Australas J Ageing*. 2019;38(2):124-131. 50. Kawashima T, Yamada S. Delirium caused by donepezil: a case study. *J Clin Psychiatry.* 2002;63(3):250-1. 51. Kenna HA, Poon AW, de los Angeles CP, Koran LM. Psychiatric complications of treatment with corticosteroids: review with case report. *Psychiatry Clin Neurosci*. 2011;65(6):549-60. 52. Kikkawa N, Sogawa R, Monji A, Sumi S, Murakawa-Hirachi T, Kubo T, Eguchi Y, Miyamoto Y, Kamo M, Tobita S, Yukawa M, Uchida R, Egoshi M, Shimanoe C. Delirium risk of histamine-2 receptor antagonists and proton pump inhibitors: A study based on the adverse drug event reporting database in Japan. *Gen Hosp Psychiatry*. 2021;72:88-91. 53. Kobayashi R, Morioka D, Suzuki A, Kawakatsu S, Otani K. Low-dose zolpidem-induced visual hallucinations in prodromal dementia with Lewy bodies. *Asian J Psychiatr.* 2021;66:102908. 54. Kobayashi T, Miyata Y, Okamoto S, Kato S. Musical hallucinations induced by bromocriptine.*Psychogeriatrics*. 2004;4(3):102-106. 55. Kohen I. Oseltamivir-induced delirium in a geriatric patient. *Int J Geriatr Psychiatry*. 2007;22(9):935-6. 56. Kolanowski A, Fick D, Waller JL, Ahern F. Outcomes of antipsychotic drug use in community-dwelling elders with dementia. *Arch Psychiatr Nurs*. 2006;20(5):217-25 57. Korkatti-Puoskari N, Tiihonen M, Caballero-Mora MA, Topinkova E, Szczerbińska K, Hartikainen S; on the Behalf of the EuGMS Task & Finish group on FRIDs. Therapeutic dilemma's: antipsychotics use for neuropsychiatric symptoms of dementia, delirium and insomnia and risk of falling in older adults, a clinical review. *Eur Geriatr Med*. 2023;14(4):709-720. 58. Lally L, McCarthy G, Meehan K. Hyperactive delirium following administration of intra-articular corticosteroid. *BMJ Case Rep*. 2017;2017:bcr2016217483. 59. Lange PW, Turbić A, Soh CH, Clayton-Chubb D, Lim WK, Conyers R, Watson R, Maier AB. Melatonin does not reduce delirium severity in hospitalized older adults: Results of a randomized placebo-controlled trial. J Am Geriatr Soc. 2024;72(6):1802-1809. 60. Lauretani F, Bellelli G, Pelà G, Morganti S, Tagliaferri S, Maggio M. Treatment of Delirium in Older Persons: What We Should Not Do! *Int J Mol Sci*. 2020;21(7):2397. 61. Lavon O, Bejel S. Safety of brotizolam in hospitalized patients. *Eur J Clin Pharmacol*. 2018;74(7):939-943. 62. Lim CJ, Trevino C, Tampi RR. Can olanzapine cause delirium in the elderly? *Ann Pharmacother.* 2006;40(1):135-8. 63. Ma SP, Tsai CJ, Chang CC, Hsu WY. Delirium associated with concomitant use of duloxetine and bupropion in an elderly patient. *Psychogeriatrics*. 2017;17(2):130-132. 64. Maclaskey D, Buchholz M, Hodges K, Bourgeois JA. Case Report: Delirium Associated With Excessive Consumption of Nicotine Gum. *J Clin Psychopharmacol*. 2022;42(3):327-329. 65. Maddalena S, Magistri C, Mellini C, Sarli G. Aripiprazole for treating delirium: A systematic review-Is it a valid yet understudied treatment? J Psychopharmacol. 2024;38(6):507-514. 66. Mancano MA. Telaprevir-Related Dermatitis; Neuroleptic Malignant Syndrome with Risperidone Long-Acting Injection; Tigecycline-Related Pancreatitis; Venlafaxine-Related Psychosis; Neurologic Adverse Effects of Ranolazine. *Hospital Pharmacy*. 2013;48(5):360-365. 67. Marvanova M. Drug-induced cognitive impairment: Effect of cardiovascular agents. *Ment Health Clin*. 2016;6(4):201-206. 68. Moellentin D, Picone C, Leadbetter E. Memantine-induced myoclonus and delirium exacerbated by trimethoprim. *Ann Pharmacother*. 2008;42(3):443-7. 69. Mollazadeh-Moghaddam K, Jamali A, Adili-Aghdam F, Akhondzadeh S. Delirium associated with donepezil in a patient with Alzheimer's disease: a case report. *Iran J Psychiatry.* 2013;8(1):59-60. 70. Monastero R, Camarda C, Pipia C, Camarda R. Visual hallucinations and agitation in Alzheimer's disease due to memantine: report of three cases. *J Neurol Neurosurg Psychiatry*. 2007;78(5):546. 71. Morikawa M, Kishimoto T. Probable dementia with Lewy bodies and risperidone-induced delirium. *Can J Psychiatry*. 2002;47(10):976. 72. Moss JM, Kemp DW, Brown JN. Combination of inhaled corticosteroid and bronchodilator-induced delirium in an elderly patient with lung disease. *J Pharm Pract.*;27(1):79-83. 73. Muralee S, Bober D, Tampi R. Delirium From the COX-2 inhibitor refecoxib. *Psychosomatics*. 2004;45(4):361-3. 74. Nasiruddin M, Fayazuddin M, Zahid M, Iftekhar S. Acute delirium in an elderly woman following zoledronate administration. *J Pharmacol Pharmacother*. 2014;5(3):217-9. 75. Nishtala PS, Chyou TY. Risk of delirium associated with antimuscarinics in older adults: A case-time-control study. *Pharmacoepidemiol Drug Saf*. 2022;31(8):883-891. 76. Noyan MA, Elbi H, Aksu H. Donepezil for anticholinergic drug intoxication: a case report. *Prog Neuropsychopharmacol Biol Psychiatry*. 2003;27(5):885-7. 77. Patten SB, Neutel CI. Corticosteroid-induced adverse psychiatric effects: incidence, diagnosis and management. *Drug Saf.* 2000;22(2):111-22. 78. Pharmacotherapy has a potential role in both the prevention and treatment of drug-induced delirium. *Drugs Ther Perspect. 2012;*28, 22–26. 79. Pisani MA, Murphy TE, Araujo KL, Van Ness PH. Factors associated with persistent delirium after intensive care unit admission in an older medical patient population. *J Crit Care.* 2010;25(3):540.e1-7. 80. Pozo ED, Arana-Asensio E, García-López P. Acute Confusional Syndrome Induced by Moxifloxacin in an Elderly Man. *J Am Geriatr Soc*. 2015;63(12):2647-2648. 81. Prado E, Paholpak P, Ngo M, Porter V, Apostolova LG, Marrocos R, Ringman JM. Agitation and psychosis associated with dementia with lewy bodies exacerbated by modafinil use. *Am J Alzheimers Dis Other Demen*. 2012;27(7):468-73. 82. Reisinger M, Reininghaus EZ, Biasi J, Fellendorf FT, Schoberer D. Delirium-associated medication in people at risk: A systematic update review, meta-analyses, and GRADE-profiles. *Acta Psychiatr Scand*. 2023;147(1):16-42. 83. Ridha BH, Josephs KA, Rossor MN. Delusions and hallucinations in dementia with Lewy bodies: worsening with memantine. *Neurology.* 2005;65(3):481-2. 84. Sadlonova M, Beach SR, Funk MC, et al. Risk Stratification of QTc Prolongation in Critically Ill Patients Receiving Antipsychotics for the Management of Delirium Symptoms. *Journal of Intensive Care Medicine*. 2023; 8850666231222470 85. Sadlonova M, Duque L, Smith D, Madva EN, Amonoo HL, Vogelsang J, Staton SC, von Arnim CAF, Huffman JC, Celano CM. Pharmacologic treatment of delirium symptoms: A systematic review. *Gen Hosp Psychiatry*. 2022;79:60-75. 86. Sagawa R, Akechi T, Okuyama T, Uchida M, Furukawa TA. Etiologies of delirium and their relationship to reversibility and motor subtype in cancer patients. *Jpn J Clin Oncol*. 2009;39(3):175-82. 87. Satyanarayana S, Campbell B. Gatifloxacin-induced delirium and psychosis in an elderly demented woman. *J Am Geriatr Soc*. 2006;54(5):871. 88. Seo SW, Suh MK, Chin J, Na DL. Mental confusion associated with scopolamine patch in elderly with mild cognitive impairment (MCI). *Arch Gerontol Geriatr*. 2009;49(2):204-207. 89. Stollings JL, Boncyk CS, Birdrow CI, Chen W, Raman R, Gupta DK, Roden DM, Rivera EL, Maiga AW, Rakhit S, Pandharipande PP, Ely EW, Girard TD, Patel MB. Antipsychotics and the QTc Interval During Delirium in the Intensive Care Unit: A Secondary Analysis of a Randomized Clinical Trial. JAMA Netw Open. 2024;7(1):e2352034. 90. Štuhec M. Auditory hallucinations associated with nitrofurantoin use: case report and review of the literature. *Wien Klin Wochenschr*. 2014;126(17-18):549-52. 91. Sugiyama Y, Tanaka R, Sato T, Sato T, Saitoh A, Yamada D, Shino M. Incidence of Delirium With Different Oral Opioids in Previously Opioid-Naive Patients. *Am J Hosp Palliat Care*. 2022; 39(10):1145-1151. 92. Suzuki Y, Saito M, Someya T. Delirium associated with duloxetine in a depressed patient with Alzheimer's dementia. *Psychiatry Clin Neurosci*. 2012;66(2):166. 93. Tanaka R, Ishikawa H, Sato T, Shino M, Matsumoto T, Mori K, Omae K, Osaka I. Incidence of Delirium Among Patients Having Cancer Injected With Different Opioids for the First Time. *Am J Hosp Palliat Care*. 2017;34(6):572-576. 94. Takeuchi N, Makino T, Nishihara M. Delirium with visual hallucinations induced by low-dose olanzapine. *Psychogeriatrics*. 2022;22(3):415-416. 95. Tomlinson EJ, Schnitker LM, Casey PA. Exploring Antipsychotic Use for Delirium Management in Adults in Hospital, Sub-Acute Rehabilitation and Aged Care Settings: A Systematic Literature Review. Drugs Aging. 2024;41(6):455-486. 96. Tsai MC, Chang PT, Yang CH, Liu ME. The Delirium Related to Oral Paliperidone in Dementia: A Case Report. *J Clin Psychopharmacol*. 201636(2):184-5. 97. Tsai PH, Chen HC, Liao SC, Tseng MC, Lee MB. Recurrent escitalopram-induced hyponatremia in an elderly woman with dementia with Lewy bodies. *Gen Hosp Psychiatry.* 2012;34(1):101.e5-7. 98. Van Rompaey B, Schuurmans MJ, Shortridge-Baggett LM, Truijen S, Bossaert L. Risk factors for intensive care delirium: a systematic review. *Intensive Crit Care Nurs*. 2008;24(2):98-107. 99. Wang D, Liu Z, Zhang W, Zu G, Tao H, Bi C. Intravenous infusion of dexmedetomidine during the surgery to prevent postoperative delirium and postoperative cognitive dysfunction undergoing non-cardiac surgery: a meta-analysis of randomized controlled trials. Eur J Med Res. 2024;29(1):239. 100. Webber C, Watt CL, Bush SH, Lawlor PG, Talarico R, Tanuseputro P. The occurrence and timing of delirium in acute care hospitalizations in the last year of life: A population-based retrospective cohort study. *Palliat Med*. 2020;34(8):1067-1077. 101. Westermeyer J, Thuras P, Yoon G, Kolla BP, Batres-Y-Carr T, Dimitropoulos E. Methadone-Related Delirium: Prevalence, Causes, and Outcomes. *J Nerv Ment Dis*. 2019;207(5):371-377. 102. Witter D, McCord M, Suryadevara U. Delirium Associated With Memantine Use in a Patient With Vascular Dementia. *J Clin Psychopharmacol*. 2015;35(6):736-7. 103. Wooster J, Cook EA, Shipman D. Psychiatric Manifestations With Sacubitril/Valsartan: A Case Report. *J Pharm Pract*. 2020;33(4):553-557. 104. Zaal IJ, Devlin JW, Hazelbag M, Klein Klouwenberg PM, van der Kooi AW, Ong DS, Cremer OL, Groenwold RH, Slooter AJ. Benzodiazepine-associated delirium in critically ill adults. *Intensive Care Med*. 2015;41(12):2130-7. 105. Zetin M. Psychopharmacohazardology: major hazards of the new generation of psychotherapeutic drugs. *Int J Clin Pract*. 2004;58(1):58-68. 106. Zheng X, Jiang H, Xue L, Qiu F, Zhu S, Li X. Delirium induced by tigecycline treatment for Acinetobacter baumannii infection: A case report. *Medicine (Baltimore)*. 2019;98(19):e15399. |

***Supplementary Figure S2.:*** *Reporting frequency of the drugs associated with an increased risk of delirium in patients >18 years (without dementia) within each of the most frequently mentioned drug classes across all included publications (n=106)*

***
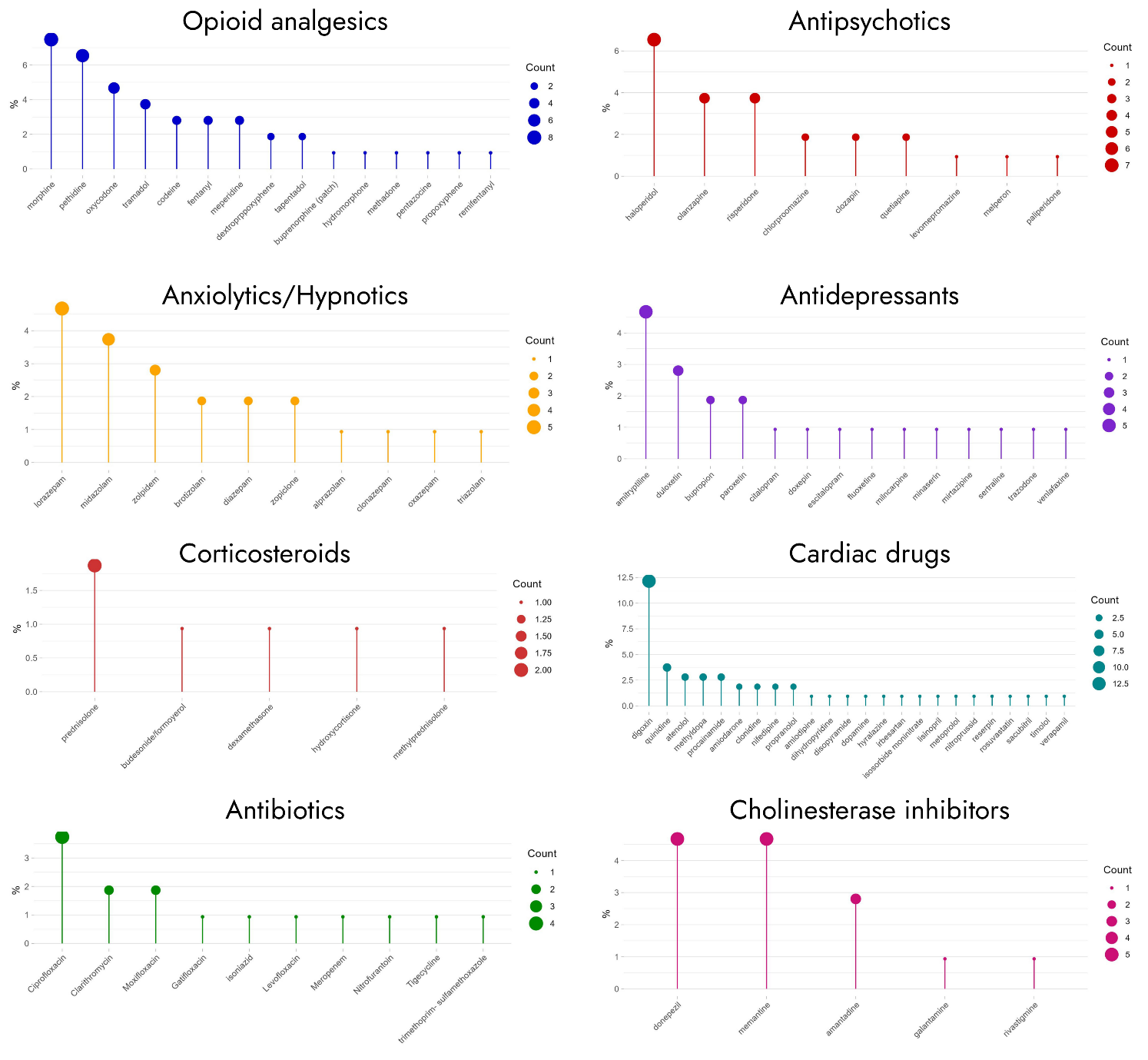
***

***Supplementary Table S4:*** *Summary of reported drug specific mechanism, dosing and symptom information for the less frequently mentioned mentioned drugs & drug classes in relation to medication risk associated with delirium in dementia patients. Only drugs for which enough information was available were include.*

| Drug class | Drug  [ATC Code] | Mechanism of action | CYP  p450 | Dose | Symptom | - Comments | REF |
| --- | --- | --- | --- | --- | --- | --- | --- |
| Cholinesterase inhibitors | **General**  **Information** | **--** | -- | -- | -- | - Even CNS-active drugs that are appropriately used in this population can accumulate in amounts that lead to problems such as delirium, sedation and falls. | S28 |
|  | Rivastigmine  [N06DA03] | Inhibits both acetylcholinesterase and butyrylcholinesterase | Not metab. via CYP | Transdermal treatment | Aggressive anxiety,  visual hallucination,  delusion | - Very prevalent in causing delusions in dementia patients (12.04%) | S5  S90 |
|  | Galantamine [N06DA04] | A reversible, competitive AChI and an allosteric modulator of nicotinic acetylcholine receptors. | CYP2D6  substrat | Half-life is about 6 hours. Complete galantamine  elimination (5 half-lives) will require approximately 30  hours. | Agitation,  dizziness | - Discontinuation - Could lead to a withdrawal syndrome | S29 |
|  | Donepezil  [N06DA02] | Donepezil selectively and reversibly inhibits the acetylcholinesterase enzyme, which breaks down acetylcholine. | CYP2D6  substrat | 5mg/day | Agitation  confusion | - Donepezil can directly cause or contribute to the onset of delirium | S50  S69  S76 |
|  | Memantine  [N06DX01] | Acetylcholine and serotonin receptors antagonist and dopamine D2 receptors agonist | Not metab. via CYP | -- | De novo visual hallucinations,  agitation,  delusion | - Patient with vascular dementia developed delirium after initiation of memantine - Discontinuation usually resolves the symptoms - Causes delusion in dementia patients in 6,28% of patients | S5  S62  S68  S70  S83  S102 |
| Dopamine Agonists | **General**  **Information** | The majority of dopamine agonists used in Parkinson disease are D2 dopamine receptor agonists | -- | - | Delusion | - Dopaminergic substances - Drugs responsible for acute confusional state - Uncertain risk for delirium - Amantadine, carbidopa, levodopa, and pramipexol are most prevalently associated with delusions | S78  S19  S43  S5 |
|  | **Amantadine**  **[**N04BB01] | Inhibit D2 dopamine receptors and N-methyl-D-aspartate (NMDA)-type glutamate receptor | Not metab. via CYP | -- | Delusion | - Highest disproportional association between delusion & amantadine | S5 |
| Antibiotics | **General**  **Information** | -- | -- | -- | Delusion | - Antibiotics are listed as risk factor for delirium superimposed on dementia. - Levofloxacin causes delusion in 8% of dementia patients. - Hallucination, disorientation 2 days after starting NF-treatment with nitrofurantoin | S37  S90  S5 |
| Analgaesic | **General**  **Information** | -- | -- | Slow-release fentanyl & morphine  Oxycodone short half-life, few drug–drug interactions and a more predictable dose–response relationships than other opiates | Agitation  Hallucination  Delusion | - Dementia is a strong risk factor for opioid-related delirium. - inpatients taking opioid drugs had a two-fold increased risk of developing delirium. - Pethidine was associated with a higher risk, and oxycodone. - Buprenorphine has fewer side effects than many other opioids, commonly used - Reports suggest that some of the nonsteroidal antiinflamatory drugs cause delirium because they have indolic moieties. These moieties are similar to serotonin, which by itself can cause delirium. | S41  S11  S10  S77 |

***Supplementary Table S5.:*** *Heat map showing at risk drug combinations (n=93) by frequency of reporting across four retrospective/prospective observation studies (total of 15546 delirium patients) by frequency of reporting (cumulative %)* [S5,S18,S49,S91].

| **Analgesic** |  |  |  |  |  |  |  |  |  |  |  |  |  |  |  |  | 33,3 |
| --- | --- | --- | --- | --- | --- | --- | --- | --- | --- | --- | --- | --- | --- | --- | --- | --- | --- |
| **Antipsychotics** |  |  |  |  |  |  |  |  |  |  |  |  |  |  |  |  | 15,1 |
| **Dopamine Agonists** | |  |  |  |  |  |  |  |  |  |  |  |  |  |  |  | 11,8 |
| **Cardiac drugs** | |  |  |  |  |  |  |  |  |  |  |  |  |  |  |  | 8,6 |
| **Antibiotics** |  |  |  |  |  |  |  |  |  |  |  |  |  |  |  |  | 7,5 |
| **Antiepileptics** | |  |  |  |  |  |  |  |  |  |  |  |  |  |  |  | 6,5 |
| **Anxiolytics/Hypnotics** | |  |  |  |  |  |  |  |  |  |  |  |  |  |  |  | 5,4 |
| **Antidepressives** | |  |  |  |  |  |  |  |  |  |  |  |  |  |  |  | 5,4 |
| **Other** |  |  |  |  |  |  |  |  |  |  |  |  |  |  |  |  | 3,2 |
| **Antiviral agents** | |  |  |  |  |  |  |  |  |  |  |  |  |  |  |  | 1,1 |
| **PPI** |  |  |  |  |  |  |  |  |  |  |  |  |  |  |  |  | 1,1 |
| **Corticosteroids** | |  |  |  |  |  |  |  |  |  |  |  |  |  |  |  | 1,1 |
|  | **Analgesic** | **Antipsychotics** | **Anxiolytics/Hypnotics** | **Antidepressives** | **Corticosteroids** | **Cardiac drugs** | **Cholinesterase inhibitors** | **Antibiotics** | **H2-receptor antagonists** | **Diuretics** | **Antihistaminika** | **Antiepileptics** | **muscle relaxants** | **PPI** | **Dopamine Agonists** | **Other** | **Cumulative Percent (%)** |
